# Maitotoxin-4, a Novel MTX Analog Produced by *Gambierdiscus excentricus*

**DOI:** 10.3390/md15070220

**Published:** 2017-07-11

**Authors:** Francesco Pisapia, Manoëlla Sibat, Christine Herrenknecht, Korian Lhaute, Greta Gaiani, Pierre-Jean Ferron, Valérie Fessard, Santiago Fraga, Silvia M. Nascimento, R. Wayne Litaker, William C. Holland, Catherine Roullier, Philipp Hess

**Affiliations:** 1 Ifremer, Phycotoxins Laboratory, rue de l’Ile d’Yeu, BP 21105, F-44311 Nantes, France; manoella.sibat@ifremer.fr (M.S.); korian.lhaute@ifremer.fr (K.L.); philipp.hess@ifremer.fr (P.H.); 2 Mer Molécules Santé (MMS) Laboratory EA2160, University of Nantes, LUNAM, Pharmacy Faculty, 9 rue Bias, F-44035 Nantes, France; christine.herrenknecht@univ-nantes.fr (C.H.); catherine.roullier@univ-nantes.fr (C.R.); 3 Department of Life Science, University of Trieste, Via Giorgieri 5, 34127 Trieste, Italy; gaiani.greta@gmail.com; 4 Toxicology of Contaminants Unit, ANSES Laboratory—French Agency for Food, Environmental and Occupational Health and Safety, Fougères, 10 B rue Claude Bourgelat, 35133 Javené, France; ferron.pj@gmail.com (P.-J.F.); valerie.fessard@anses.fr (V.F.); 5 Instituto Español de Oceanografía (IEO), Centro Oceanográfico de Vigo, Subida a Radio Faro 50, 36390 Vigo, Spain; santi.fraga@vi.ieo.es; 6 Laboratório de Microalgas Marinhas, Departamento de Ecologia e Recursos Marinhos, Universidade Federal do Estado do Rio de Janeiro (UNIRIO), Rio de Janeiro 22290-240, Brazil; silvia.nascimento@unirio.br; 7 National Oceanic and Atmospheric Administration, National Ocean Service, National Centers for Coastal Ocean Science, Center for Coastal Fisheries and Habitat Research (CCFHR), 101 Pivers Island Road, Beaufort, NC 28516, USA; wayne.litaker@noaa.gov (R.W.L.); chris.holland@noaa.gov (W.C.H.)

**Keywords:** *Gambierdiscus excentricus*, maitotoxin-4, bioguided fractionation, size-exclusion chromatography (LH-20), neuro-2a (N2a) assay, LC-HRMS/MS (Q-Tof 6550), LC-LRMS/MS (API4000 QTrap)

## Abstract

Maitotoxins (MTXs) are among the most potent toxins known. These toxins are produced by epi-benthic dinoflagellates of the genera *Gambierdiscus* and *Fukuyoa* and may play a role in causing the symptoms associated with Ciguatera Fish Poisoning. A recent survey revealed that, of the species tested, the newly described species from the Canary Islands, *G. excentricus*, is one of the most maitotoxic. The goal of the present study was to characterize MTX-related compounds produced by this species. Initially, lysates of cells from two Canary Island *G. excentricus* strains VGO791 and VGO792 were partially purified by (i) liquid-liquid partitioning between dichloromethane and aqueous methanol followed by (ii) size-exclusion chromatography. Fractions from chromatographic separation were screened for MTX toxicity using both the neuroblastoma neuro-2a (N2a) cytotoxicity and Ca^2+^ flux functional assays. Fractions containing MTX activity were analyzed using liquid chromatography coupled to high-resolution mass spectrometry (LC-HRMS) to pinpoint potential MTX analogs. Subsequent non-targeted HRMS analysis permitted the identification of a novel MTX analog, maitotoxin-4 (MTX4, accurate mono-isotopic mass of 3292.4860 Da, as free acid form) in the most toxic fractions. HRMS/MS spectra of MTX4 as well as of MTX are presented. In addition, crude methanolic extracts of five other strains of *G. excentricus* and 37 other strains representing one *Fukuyoa* species and ten species, one ribotype and one undetermined strain/species of *Gambierdiscus* were screened for the presence of MTXs using low resolution tandem mass spectrometry (LRMS/MS). This targeted analysis indicated the original maitotoxin (MTX) was only present in one strain (*G. australes* S080911_1). Putative maitotoxin-2 (p-MTX2) and maitotoxin-3 (p-MTX3) were identified in several other species, but confirmation was not possible because of the lack of reference material. Maitotoxin-4 was detected in all seven strains of *G. excentricus* examined, independently of their origin (Brazil, Canary Islands and Caribbean), and not detected in any other species. MTX4 may therefore serve as a biomarker for the highly toxic *G. excentricus* in the Atlantic area.

## 1. Introduction

**Figure 1:**
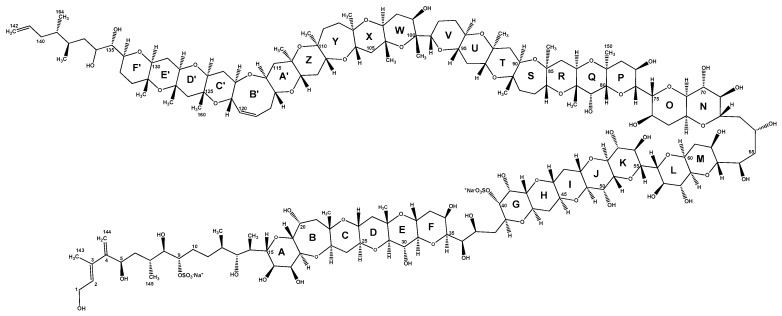
Absolute stereochemistry of maitotoxin (MTX) according to Sasaki et al. [31] and Nonomura et al. [32].

Maitotoxin (MTX) (Figure 1) is among the most potent marine toxins identified to date, with an intraperitoneal (i.p.) lethal dose 50 (LD_50_) in mice of 0.050 µg kg^−1^ [1]. Its oral potency, however, is much lower [2], probably due to low intestinal absorption caused by its high molecular weight and hydrophilicity. Consequently, MTX is primarily found in the tissues associated with the digestive tract of fish and is believed to play a role in ciguatera fish poisoning (CFP) if gut and liver tissues are consumed [3].

Maitotoxin was first detected in 1976 in the viscera of the bristletooth surgeonfish *Ctenochaetus striatus* (in Tahitian “maito”, hence its name) collected in Tahiti (French Polynesia) and it was initially suspected of contributing to the diversity of ciguatera symptoms [4,5]. Eleven years later, the toxin was isolated from the dinoflagellate *Gambierdiscus* by Yasumoto, et al. [6] confirming the source of the toxin isolated from contaminated fish. Purified MTX exists as a white amorphous solid that is soluble in polar solvents (e.g., water, methanol and dimethylsulfoxide) and it is relatively stable in alkaline but not in acidic conditions. In aqueous solution, pure MTX tends to adhere to both glass and plastic surfaces [7,8]. When dissolved in methanol-water, MTX exhibits a single UV absorbance maximum at 230 nm [9] due to the presence of a conjugated diene at one extremity of the molecule (C_2_-C_3_-C_4_-C_144_, Figure 1).

Experiments using purified MTX showed it causes a rapid influx of external Ca^2+^ and a steep increase of intracellular Ca^2+^ (_i_Ca^2+^) concentration in a wide variety of cells [10,11]. The Ca^2+^ influx elicited by MTX leads to numerous secondary events, including: depolarization in neuronal cells [12], phosphoinositide breakdown [13], smooth muscle contraction [14,15,16], induction of acrosome reaction in sperm [17,18,19], secretion of neurotransmitters (e.g., dopamine [20], noradrenaline [21,22], GABA [23]), hormones (e.g., insulin [24,25]) and inflammatory intermediates (e.g., arachidonic acid [26] and histamine [27]), formation or activation of large cytolytic/oncotic pores [28,29,30].

The complete chemical structure of MTX was elucidated in 1993 following purification from the *Gambierdiscus* strain (GII-1) isolated from Gambier Islands (French Polynesia) [1]. That analysis showed MTX is the largest non-polymeric marine toxin identified to date, consisting of a ladder-shaped cyclic polyether that is composed of 32 fused ether rings, 28 hydroxyl groups, 21 methyl groups, two sulfates and 98 chiral centers (molecular formula: C_164_H_256_O_68_S_2_Na_2_, mono-isotopic mass = 3423.5811 Da for the di-sodium salt). In 1996, the stereochemistry of the entire molecule was also assigned [31,32,33] (Figure 1). Gallimore and Spencer [34] contested the stereochemistry of the junction between J and K rings according to a mechanistic hypothesis for the biosynthesis of marine ladder-shaped polyethers. Subsequent studies by Nicolaou and Frederick [35] and Nicolaou, et al. [36] supported the originally assigned structure based on NMR spectroscopic data, computational studies and providing chemical synthesis and NMR analysis of the GHIJK ring system. X-ray crystal structure of MTX is needed to solve this controversy.

Though the effects of MTX at the cellular level are well characterized, its actual mode of action has not been fully elucidated. Initially, MTX was considered to be a specific activator of voltage-gated calcium channels [37,38,39]. In actuality, MTX increases _i_Ca^2+^ by activating a voltage-independent Ca^2+^ entry mechanism in the plasma membrane, without directly promoting the release of Ca^2+^ from intracellular storage compartments [40,41,42]. So far, MTX has been shown to activate non-selective ion channels, probably involving TRPC1 (transient receptor potential canonical 1) [40,41,43,44]. The activation of the sodium-calcium exchanger in reverse mode has also been observed in rat aortic smooth muscle cells [45]. The activation of the sodium-hydrogen exchanger equally appears to play a role in MTX cytotoxic activity in cortical neurons [46] and it may be a consequence of MTX-induced intracellular acidification, probably involving voltage-gated sodium channels [47]. Maitotoxin is also likely to convert the Ca^2+^-ATPase (PCMA) pump into a Ca^2+^-permeable non-selective ion channel, as demonstrated in PCMA-overexpressed *Spodoptera frugiperda* (Sf9) insect cells and human embryonic kidneys (HEK-293 cells) [48]. To date, it is unclear whether MTX directly interacts with any of these targets. Several research groups have postulated that MTX may bind a still undescribed MTX-receptor [35,42,49,50]. Since the specific molecular target of MTX is still unknown, its structure-activity relationship can only be inferred based on its NMR structural features and analogies with other ladder-shaped polyether toxins. Konoki, et al. [51] first hypothesized that the hydrophobic side of MTX (rings R through F’) penetrates the phospholipid bilayer of cell membranes and the hydrophilic portion of the molecule, presenting the polyhydroxy- groups and the two sulfate ester groups (rings A through Q), remains outside the cell (Figure 1). Modeling studies conducted by Reyes et al. [11] corroborate this hypothesis.

Sulfate ester groups seem to be critical for the biological activity of MTXs [52,53]. A study conducted by Murata et al. [52] in particular showed that desulfatation or hydrogenation of MTX significantly decreased its ability to induce Ca^2+^ influx or phosphoinositide breakdown in insulinoma or glioma cells. Murata, et al. [54] also hypothesized that a self-assemblage of four or more molecules could occur to form a pore on cell membranes for non-selective ion influx; however, this hypothesis has not been confirmed.

During the 1990s, two other MTX analogs, MTX2 and MTX3, were isolated by Holmes et al. [53,55]. Maitotoxin-2 (MTX2) was obtained from a single Australian *Gambierdiscus* strain from Queensland (NQ1) [55]. It had an i.p. LD_50_ in mice of 0.080 µg kg^−1^ [53], i.e., 1.6-fold less toxic than MTX [1]. When dissolved in acetonitrile-water, MTX2 had a single UV absorbance maximum at 230 nm [53], identical to that reported for MTX in methanol-water [9]. The molecular structure of MTX2 has not been elucidated yet. Lewis, et al. [56] conducted LC-LRMS analyses of material isolated from strain NQ1 in ionspray ionization in positive ion acquisition mode (IS^+^), ionspray ionization in negative ion acquisition mode (IS^−^) and fast atom bombardment ionization in negative ion acquisition mode (FAB^−^) and suggested that MTX2 is mono-sulfated with a molecular weight (*MW*) of 3298 Da (as mono-sodium salt).

Maitotoxin-3 (MTX3) was isolated from the Australian *Gambierdiscus* strain WC1/1 [53]. Maitotoxin-3 was found to be toxic in mice, inducing similar symptoms than those observed for MTX and MTX2, but scarcity of the purified compound did not permit the determination of MTX3 potency (i.p. LD_50_ in mice) [53]. On a reversed-phase column, MTX3 elutes earlier than MTX2 and later than MTX when using a linear gradient of acetonitrile/water [53]. When dissolved in acetonitrile-water, MTX3 had a UV spectrum composed of two peaks, a minor peak at 200 nm and a major peak at 235 nm, slightly higher than MTX and MTX2 [53]. Lewis et al. [56] conducted LC-LRMS analyses of material isolated from strain WC1/1 in IS^+^ acquisition mode. Their results suggested that MTX3 is di-sulfated with a *MW* = 1060.5 Da (as di-sodium salt). The actual molecular structure of MTX3 has yet to be determined.

Since the molecular structures of MTX2 and MTX3 are still unknown, the only structural feature that is known to be common to the MTX class of toxins is the presence of (at least) one sulfate ester group. Desulfatation experiments conducted by Holmes and Lewis [53] on the three MTX analogs again suggested that at least one of the sulfate ester groups is critical for the bioactivity.

A schematic summary of the properties of three MTXs known to date with relevant chemical information is listed in Table 1. A recent study conducted by Lewis et al. [57] indicated that several strains of *Gambierdiscus*/*Fukuyoa* produce multiple MTX congeners, in one case more than four (*G. belizeanus* CCMP399), suggesting broader chemical diversity than what is known so far within MTX group. These analogs are not listed here since no further information beyond activity was provided in that study.

Fifteen *Gambierdiscus* and three *Fukuyoa* species have been described in the past few decades [58,59,60,61,62,63,64,65,66]. As previously suggested [58], ongoing taxonomic studies being conducted by Tunin-Ley, et al. [67] also indicate that the biological diversity within these genera could be much higher than expected to date. The functional MTX toxicity for many of these species has been examined using erythrocyte lysis assay [68,69] or neuroblastoma SH-SY5Y Ca^2+^ assay [57]. Of the species tested, *G. excentricus*, which was described from isolates obtained in the Canary Islands, exhibited much higher maito- and cigua (CTX)-toxicity than any other species known to occur in the Atlantic [61,69]. Its MTX- and CTX-toxicity were comparable to *G. polynesiensis*, the most toxic species isolated to date from the Pacific [70,71]. Due to its high MTX-toxicity, the aim of the present study was to characterize the MTX congeners produced by *G. excentricus* and to determine if they were the same or different from those previously identified. The approach was based on bioguided fractionation of the aqueous methanol fraction containing MTXs using size-exclusion chromatography (LH-20). Individual fractions were screened for MTX activity using the N2a cytotoxicity and Ca^2+^ flux functional assays. The MTX positive fractions were subjected to chemical analyses (LC-HRMS, LC-HRMS/MS and LC-LRMS/MS) to identify potential MTX congeners. Unfractionated methanolic extracts of a total of 44 strains representing one species of *Fukuyoa* and 11 species, one ribotype and one strain of *Gambierdiscus* whose species identity has yet to be determined were also screened at the same time. The results indicated *G. excentricus* produces a novel MTX congener, MTX4, which was not found in any of the other species tested.

## 2. Results

### 2.1. Toxicity of the Aqueous Methanol Fractions (MSFs) from G. excentricus Strains VGO791 and VGO792

The toxicity of the aqueous MeOH soluble fractions (MSFs) of strains VGO791 and VGO792 from the Canary Islands were assessed using the N2a cytotoxicity assay performed at the Phycotoxins Laboratory (Ifremer, Nantes, France). The assay was calibrated using a purified MTX standard, which induced mortality of the N2a cells in a concentration-dependent manner, with an EC_50_ of 158.5 ± 5.4 (SD, *n* = 3) ng MTX mL^−1^ (Figure S1). Strain VGO791 exhibited a toxin content of 0.65 ± 0.13 ng MTX equivalents (eq) cell^−1^ and VGO792 a toxin content of 0.19 ± 0.05 ng MTX eq cell^−1^. Strain VGO791 was therefore 3.4-fold more toxic than VGO792. The results for VGO791 were in accordance with a previous study conducted by Fraga et al. [61], which estimated the toxicity of this strain at 0.60 ± 0.24 MTX eq cell^−1^. In contrast, the toxicity estimated for VGO792 in the present study was 2.5-fold lower than that of 0.48 ± 0.16 ng MTX eq cell^−1^ obtained by Fraga et al. [61].

### 2.2. Screening of Fractionated G. excentricus Extracts Using Neuroblastoma N2a Assays

#### 2.2.1. N2a Cytotoxicity Assay

**Figure 2:**
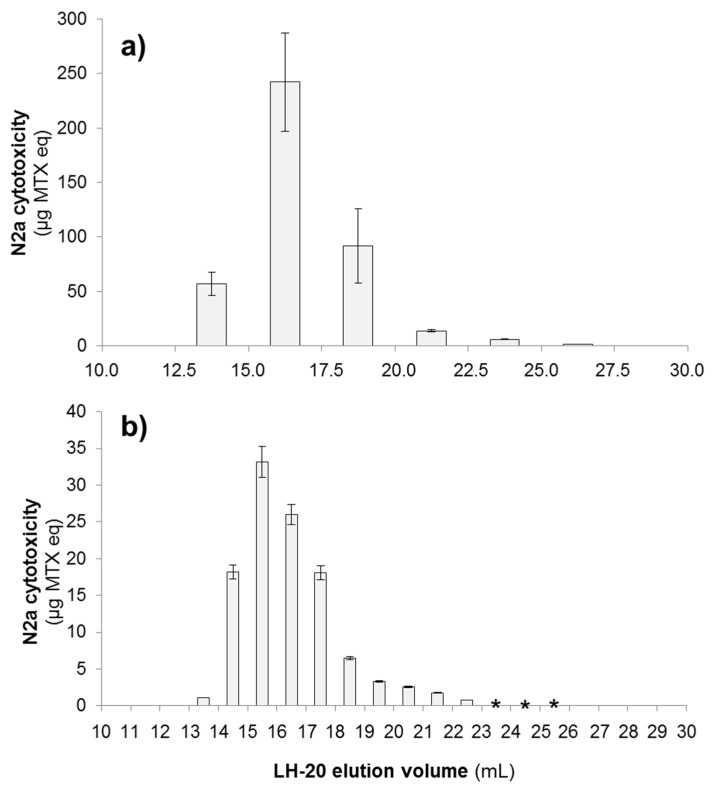
Estimated maitotoxin equivalents (µg MTX eq) in the LH-20-fractionated MSFs of *G. excentricus* strains VGO791 and VGO792 measured using the N2a cytotoxicity assay. (**a**) MTX eq of the individual 2.5 mL-fractions for *G. excentricus* VGO791 (2.20 million cells extracted in original MSF, approximately half of which was loaded on the LH-20 column). (**b**) MTX eq of the individual 1 mL-fractions for *G. excentricus* VGO792 (2.16 million cells extracted in original MSF, approximately half of which was loaded on the LH-20 column). The x-axis is expressed as the total elution volume (mL) that has passed through the LH-20 column when each fraction was collected. Error bars represent assay variability (standard deviation, SD) measured by running extracts in three separate assays. In each assay, three separate wells were used for assaying each fraction. Cytotoxicity was observed only in LH-20 fractions within an elution volume (*V*_e_) range of (**a**) *V*_e_ = 12.5–27.5 mL and (**b**) *V*_e_ = 13.0–26.0 mL. Asterisks (*) in (**b**) indicate detection of non-quantifiable cytotoxicity in fractions with *V*_e_ = 23.0–26.0 mL. Fractions corresponding to *V*_e_ < 10.0 mL and *V*_e_ > 30.0 mL are not shown because no cytotoxicity was observed in those fractions.

The fractionation of the extracted MSF sample from strain VGO791 was accomplished using size-exclusion chromatography (SEC, LH-20). The fractions containing MTX activity consistently eluted within an elution volume (*V*_e_) range of 12.5–27.5 mL. The most toxic fractions were found in *V*_e_ = 15.0–17.5 mL (up to 58.8% total cytotoxicity) (Figure 2a). Similarly, toxic fractions from strain VGO792 eluted in the range of *V*_e_ = 13.0–26.0 mL, with the most toxic fractions being fraction *V*_e_ = 15.0–16.0 mL (up to 29.8% total cytotoxicity) and *V*_e_ = 16.0–17.0 mL (up to 23.3% total cytotoxicity) (Figure 2b). Fractions of VGO792 corresponding to *V*_e_ = 23.0–26.0 mL showed slight cytotoxic activity (N2a cell survival ~80–90%) only when the highest concentration of cell extracts was tested (150 *Gambierdiscus* cell eq per well). Serial dilutions of the latter fractions contained no detectable toxicity as measured using the N2a assay (N2a cell survival >90%). Consequently, sigmoidal dose-response curves could not be plotted and EC_50_ values could not be calculated for quantification purposes for the low toxicity fractions.

The N2a cytotoxicity assay showed that toxic compound(s) eluted right after the total exclusion volume of the LH-20 column (i.e., approximately 30% of bed volume, 12.4 mL). LH-20 chromatography separates compounds with *MW* ≤ 5000 Da according to their size (i.e., smaller compounds elute later than bigger ones) meaning the toxic compound(s) from these strains are likely to fall in the range of ~3000–3500 Da, consistent with the molecular weight of MTX.

#### 2.2.2. N2a Calcium Flux Assay

The N2a-based high-content screening (HCS) assay for calcium (Ca^2+^) flux was performed at the ANSES Laboratory (Fougères, France). A Ca^2+^ flux assay was used in this study to measure changes in internal Ca^2+^ concentration in N2a cells. The assay works by loading cells with a fluorescent dye, in this case Fluo-4-AM, whose fluorescence changes as a function of intracellular Ca^2+^ (_i_Ca^2+^) concentration. Maitotoxin standard elicited an increase of _i_Ca^2+^ in N2a cells in a concentration-dependent manner (Figure S2). Since MTX induces influx of Ca^2+^ into cells, a significant increase in fluorescence is consistent with the presence of MTX (Section 4.5.2). Results were expressed as a fold of intensity compared to control treatment (5% MeOH in FCS-free N2a medium).

**Figure 3:**
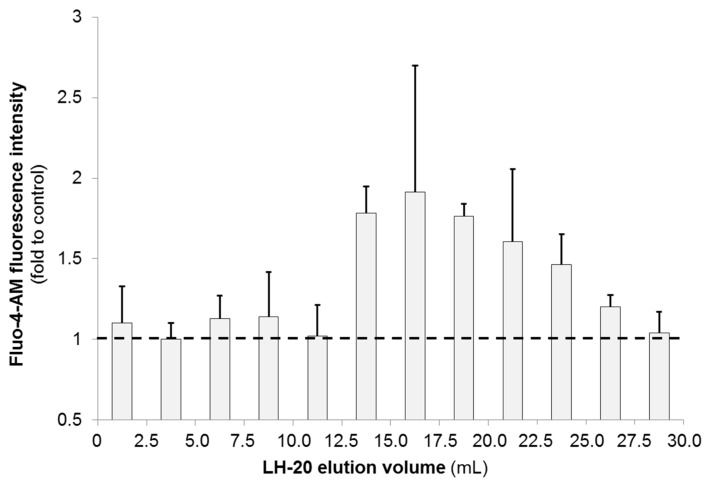
Calcium (Ca^2+^) influx into N2a cells induced by LH-20 fractions of *G. excentricus* VGO791 (MSF sample). Ca^2+^ flux was measured using fluorescence of Fluo-4-AM (488 nm). Fluo-4-AM fluorescence was expressed as a fold of intensity compared to the control wells (horizontal dashed line). Error bars represent assay variability (standard deviation, SD) measured by running extracts in three separate assays. In each assay, three separate wells were used for assaying each fraction. Ca^2+^ influx was observed only in LH-20 fractions corresponding to an elution volume (*V*_e_) of 12.5–27.5 mL. Fractions eluting after a *V*_e_ of 30 mL were omitted because they exhibited no MTX-induced fluorescence changes.

The crude extract (CE) and the MSF of *G. excentricus* VGO791 increased _i_Ca^2+^ levels in N2a cells up to the saturation level (data not shown), indicating the presence of compounds exhibiting activity similar to MTX. Only the six LH-20 fractions of MSF falling within a *V*_e_ range of 12.5–27.5 mL showed an increase in _i_Ca^2+^ levels of between 1.2- and 1.8-fold (Figure 3), suggesting that MTX-related compound(s) mostly eluted in these fractions. These findings are consistent with the results obtained by the N2a cytotoxicity assay (Section 2.2.1).

### 2.3. Liquid Chromatography Coupled to Full-Scan High Resolution Mass Spectrometry and Discovery of a New Maitotoxin Analog

**Figure 4:**
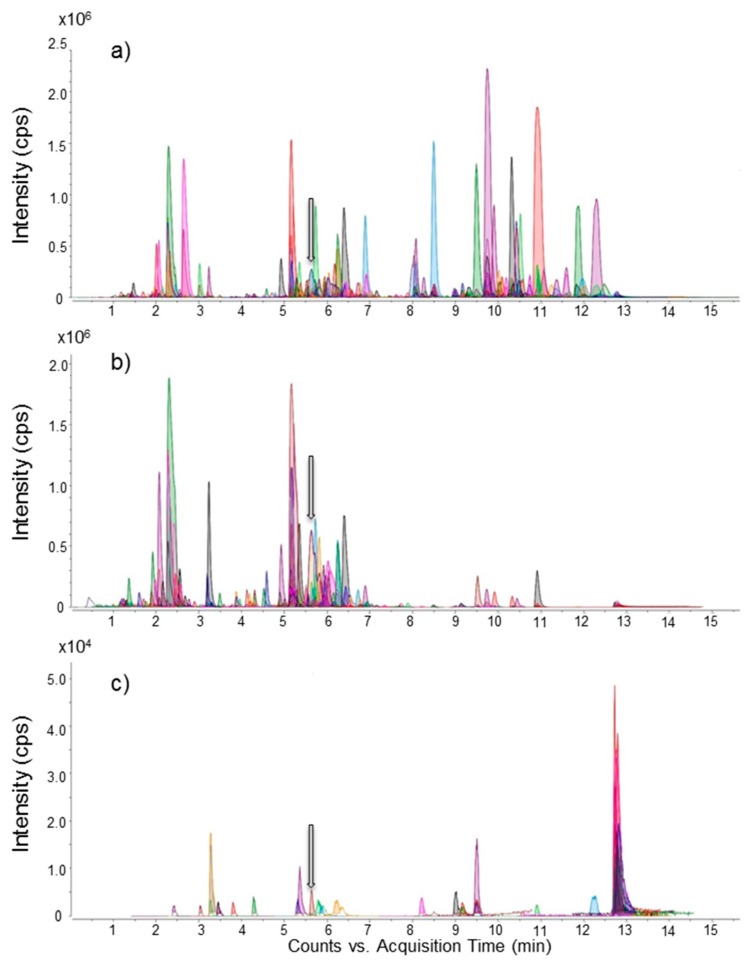
Negative Extracted Compound Chromatograms (ECCs) of *G. excentricus* VGO791 samples: (**a**) crude extract; (**b**) aqueous methanol soluble fraction (MSF); (**c**) the most toxic LH-20 fraction (*V*_e_ = 15.0–17.5 mL) of MSF. The different color peaks represent unique features. The highlighted grey arrow indicates the peak corresponding to maitotoxin-4 (MTX4).

The Molecular Feature Extraction (MFE) algorithm of the Agilent MassHunter Qualitative Analysis software allows for retreatment of raw data of non-targeted HRMS analysis (Q-Tof 6550). Extracted compound chromatograms acquired in negative ionization mode (negative ECCs) of *G. excentricus* VGO791 confirmed that liquid-liquid partitioning and size-exclusion chromatography purification steps considerably reduced data complexity. More than one thousand features were detected in the crude extract, while only 40 features (3%) were present in the most toxic LH-20 fraction (*V*_e_ = 15.0–17.5 mL) (Figure 4). Similarly, MFE results for *G. excentricus* VGO792 also exhibited a reduction of data complexity from more than five thousands features to only 239 (4.3%) in the most toxic LH-20 fraction of MSF (*V*_e_ = 15.0–16.0 mL), data not shown. These results indicate that fractionation via liquid-liquid partitioning and size-exclusion chromatography (LH-20) is a suitable strategy for *Gambierdiscus* toxin purification. In particular, LH-20 is an efficient clean-up step for high molecular weight compounds as it allows for sufficient purification for individual compounds to be identified as potential MTX congeners.

Among the occurring negative ions present in the negative mode ECCs of the most toxic LH-20 fractions of *G. excentricus* VGO791 and VGO792, only one bi-charged anion presented, like MTX, an *m*/*z* ratio >1500. This compound possesses a retention time close to that of MTX (Δ*RT* = +0.49 min). Interestingly, it was detected only in the most toxic LH-20 fractions of both strains (as well as in their MSF and crude extract) and was not detected in non-toxic fractions. Thus, the presence of this compound in toxic fractions only, along with similar MS and chromatographic behavior as MTX, suggested that the compound could be a novel MTX analog and hence it was named maitotoxin-4 (MTX4).

### 2.4. Mass Spectral Comparison of Maitotoxin-4 with Maitotoxin

#### 2.4.1. Negative Mode HRMS Spectra

Maitotoxin-4 (MTX4) had a spectral profile similar to MTX. Maitotoxin-4 spectra originate from the pre-purified LH-20 fraction (*V*_e_ = 15.0–16.0 mL) of *G. excentricus* VGO792. The assigned negative HRMS ion species (accurate mono-isotopic *m*/*z*) for MTX and MTX4 are listed in Table 2.

**Figure 5:**
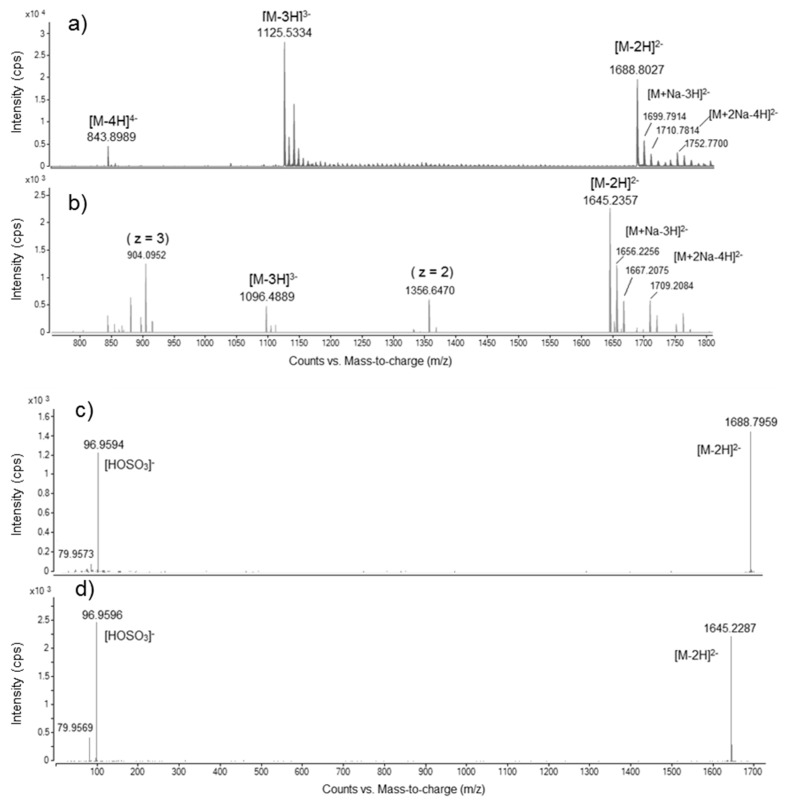
Raw spectra in full scan, negative ion acquisition mode HRMS of (**a**) maitotoxin (MTX) and (**b**) maitotoxin-4 (MTX4) acquired over an *m*/*z* range from 100 to 3200, focused on the *m*/*z* range from 800 to 1800. Maitotoxin-4 spectra originate from the pre-purified LH-20 fraction (*V*_e_ = 15.0–16.0 mL) of *G. excentricus* VGO792. Note the presence of bi-charged and tri-charged ion clusters for both MTX and MTX4. Negative mode ESI product ion spectra of bi-charged molecular anions of (**c**) MTX and (**d**) MTX4 at an average of three collision energies (CE): 50, 100 and 200 eV over an *m*/*z* range from 25 to 3200, focused on the *m*/*z* range from 25 to 1700. Note the same product ion [HOSO_3_]^−^ for both MTX and MTX4. Nota bene: the *m*/*z* values highlighted in the figure correspond to the accurate, i.e., measured mono-isotopic *m*/*z*.

Both MTX and MTX4 presented: (i) bi-charged molecular anions [M − 2H]^2−^, with accurate mono-isotopic *m*/*z* of 1688.8027 for MTX (Δppm: −0.8) and 1645.2357 for MTX4 and (ii) tri-charged molecular anions [M − 3H]^3−^, with accurate mono-isotopic *m*/*z* of 1125.5334 for MTX (Δppm: −1.4) and 1096.4889 for MTX4 (Figure 5a,b). Hence, MTX4 presents a lower mass than the MTX standard: 3292.4860 (MTX4 accurate calculated mass, free acid form) versus 3379.6172 (MTX theoretical exact mass, free acid form), ΔM = 87.1312. For MTX only, it was also possible to observe the quadri-charged molecular anion [M − 4H]^4−^, with accurate mono-isotopic *m*/*z* of 843.8989 (Δppm: −2.1) (Figure 5a). Further, the following adducts (accurate mono-isotopic *m*/*z*) could be assigned: [M + Na − 3H]^2−^ = 1699.7914 for MTX (Δppm: +0.5) and 1656.2256 for MTX4; [M + 2Na − 4H]^2−^ = 1710.7814 for MTX (Δppm: +1.1) and 1667.2075 for MTX4 (Figure 5a,b). In the negative mode HRMS spectrum of MTX4, two additional peaks were observed, with accurate mono-isotopic *m*/*z* of 1356.6470 (z = 2, bi-charged anion) and 904.0954 (z = 3, tri-charged anion), suggesting either that some fragmentation occurred in the ESI source or that co-elution occurred during the chromatographic separation (Figure 5b).

#### 2.4.2. Negative Mode HRMS/MS Spectra

Maitotoxin-4 (MTX4) was further analyzed using Collision Induced Dissociation in Q-Tof targeted MS/MS mode (HRMS/MS). Maitotoxin-4 spectra originate from the pre-purified LH-20 fraction (*V*_e_ = 15.0–16.0 mL) of *G. excentricus* VGO792. Accurate mass data and isotopic distributions for the precursor and product ions of MTX4 were compared to spectral data of the reference compound, MTX, obtained in identical experimental conditions.

HRMS/MS fragmentation of the bi-charged molecular anion ([M − 2H]^2−^) of MTX and MTX4 showed that the two molecules share the same product ion at an average *m*/*z* of 96.9593 ± SD 0.0003 (*n* = 4) when using a collision energy (CE) ≥ 100 eV (Figure 5c,d). This fragment corresponds to the hydrogenated sulfate anion [HOSO_3_]^−^ (Δppm: +8.3). No other characteristic fragment ion has been found at any of the collision energies tested.

### 2.5. Quantification of MTX4 via LC-LRMS/MS

The limit of detection (LOD) and the limit of quantification (LOQ) of the MRM transition [M − 2H]^2−^ → [M − 2H]^2−^ of MTX chosen for quantification purpose, were, respectively, 0.64 µg mL^−1^ and 2.12 µg mL^−1^. LOD and LOQ of MTX4 were assumed being the same of MTX. The LOD and the LOQ of the MRM transition [M − 3H]^3−^ → [M − 3H]^3−^ of MTX were, respectively, 0.12 µg mL^−1^ and 0.39 µg mL^−1^. As shown in Figure 5a, the tri-charged molecular anion of MTX gave a more intense response than the bi-charged molecular anion in our analytical conditions. Nevertheless, that was not the case for MTX4 (Figure 5b). Although the higher LOD and LOQ, the MRM transition [M − 2H]^2−^ → [M − 2H]^2−^ was chosen for quantification instead of the MRM transition [M − 3H]^3−^ → [M − 3H]^3−^.

Table 3 presents the results of (i) the amount of MTX4 present in crude extracts from approximately 2.2 million cells of *G. excentricus* VGO791 and VGO792 and (ii) the amount of MTX4 remaining after the subsequent liquid-liquid partitioning and LH-20 chromatography purification steps. The quantities of MTX4 were estimated using multiple reaction monitoring (MRM) mode on LC-LRMS/MS as described in Section 4.6.2. The amounts of MTX4 present were expressed as µg MTX eq, assuming an equimolar response of MTX4 and MTX in MS. The efficiency of each purification step was expressed in percent recovery relative to the amount of MTX in the crude extract. The combination of the two purification steps allowed for a percent recovery of 72% for VGO791 and 70% for VGO792 if all the toxic fractions with a concentration of MTX4 > LOQ were combined (Table 3).

### 2.6. Relationship between N2a Cytotoxicity and MTX4 Content

The MTX contents of MSFs estimated via N2a cytotoxicity assay were: 1427 ± 289 µg MTX eq for *G. excentricus* VGO791 (2.20 million cells) and 408 ± 110 µg MTX eq for *G. excentricus* VGO792 (2.16 million cells). This estimation is, respectively, 10.4- and 11.8-fold higher than the MTX4 content estimated via LC-LRMS/MS (137.4 and 34.2 µg MTX eq, respectively, Section 2.5).

**Figure 6:**
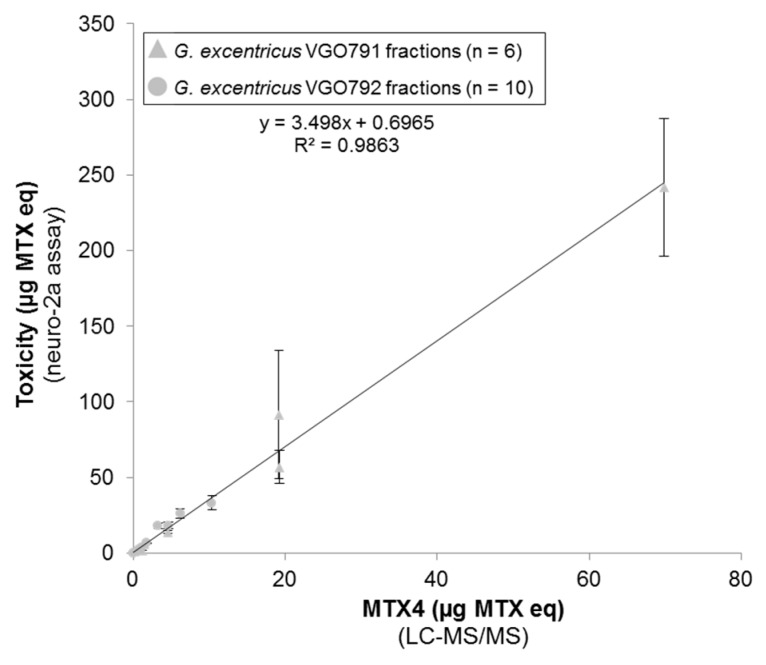
Linear correlation between the MTX equivalents (N2a cytotoxicity assay) and the MTX4 content (LC-LRMS/MS) in all LH-20 fractions containing quantifiable amounts of the two strains of *G. excentricus* VGO791 (*n* = 6) and VGO792 (*n* = 10). Results are expressed in µg MTX eq, assuming an equimolar response of MTX4 and MTX in MS. Error bars represent assay variability (standard deviation, SD) measured by running extracts in three separate assays. In each of these assays, three separate wells were used for assaying each fraction. The MTX4 LC-LRMS/MS determinations were not replicated.

Six LH-20 fractions of VGO791 (*V*_e_ = 12.5–27.5 mL) and ten LH-20 fractions of VGO792 (*V*_e_ = 13.0–23.0 mL) contained measurable quantities of (i) MTX equivalent toxicity (µg MTX eq, starting with an initial crude extract of ~2.2 million cells) as measured using the N2a cytotoxicity assay (Section 2.2.1) and (ii) MTX4 (µg MTX eq) using LC-LRMS/MS (Section 2.5). Toxin content estimated with the N2a cytotoxicity assay was plotted against MTX4 quantification performed using LC-LRMS/MS. The linear correlation between the amount of MTX equivalents (N2a cytotoxicity assay) and the MTX4 content (LC-LRMS/MS) among the 16 toxic LH-20 fractions (slope: 3.498, *R*^2^: 0.986, *n* = 16) suggests that MTX4 is a major contributor agent for the MSF-toxicity of both *G. excentricus* strains (Figure 6).

### 2.7. Diversity of MTX Analogs among Gambierdiscus and Fukuyoa Strains

A total of two strains of *Fukuyoa* (one species, *F. ruetzleri*) and 42 strains of *Gambierdiscus* representing 11 species, one ribotype and one strain of *Gambierdiscus* whose identity has yet to be determined, were screened for the presence of MTX analogs using the LC-LRMS/MS conditions described in Section 4.6.2.

The LC-LRMS/MS method consisted of searching for several MRM transitions of different parent ions pointing towards the hydrogenated sulfate anion [HOSO_3_]^−^ (96.9 *m*/*z*). The parent ions for MTX and MTX4 were chosen from the negative ionization mode HRMS analysis conducted in this study (Section 2.4.1). More precisely, the third isotopic peak (M + 2) was chosen because it was the most intense (i.e., 1689.8 *m*/*z* and 1126.2 *m*/*z* for MTX and 1645.2 *m*/*z* and 1097.1 *m*/*z* for MTX4). In the absence of MS/MS data on MTX2 and MTX3, parent ions were selected from the literature [56,72]. For MTX2, bi-charged and tri-charged adducts with Na^+^ and K^+^ have been reported in a previous study [56] and these have been used for LRMS/MS analysis. Furthermore, the bi-charged and tri-charged molecular anions have been searched assuming similar MS behavior of MTX4 compared to MTX. Thus, both molecular ([M − 2H]^2−^ and [M − 3H]^3−^) and pseudomolecular anions (i.e., Na^+^ and K^+^ adducts) have been searched for. Maitotoxin-3 is assumed to be di-sulfated [56] and was tentatively detected in previous studies using the loss of sulfate from the mono-charged mono-sodium adduct, i.e., the MRM transition [M + Na − 2H]^−^ → [HOSO_3_]^−^ (*m*/*z* 1037.6 → 96.9) [59,64,66,71,72,73,74]. With the aim of increasing selectivity, in this study the mono-charged and bi-charged molecular anions, as well as the mono-charged di-sodium adduct have been searched for, assuming similar MS behavior as that of MTX and MTX4 (Section 4.6.2).

Results are summarized in Table 4. Limits of detection (LODs) in Table 4 have been calculated from the number of cells extracted, specified in Table 5. Maitotoxin was found only in one strain, *G. australes* S080911_1, at a concentration of 22.6 ± 0.5 pg MTX cell^−1^. Maitotoxin-4 was found in all seven *G. excentricus* strains examined (13–72.8 pg MTX eq cell^−1^), independently of their geographical origin (Table 4).

**Figure 7:**
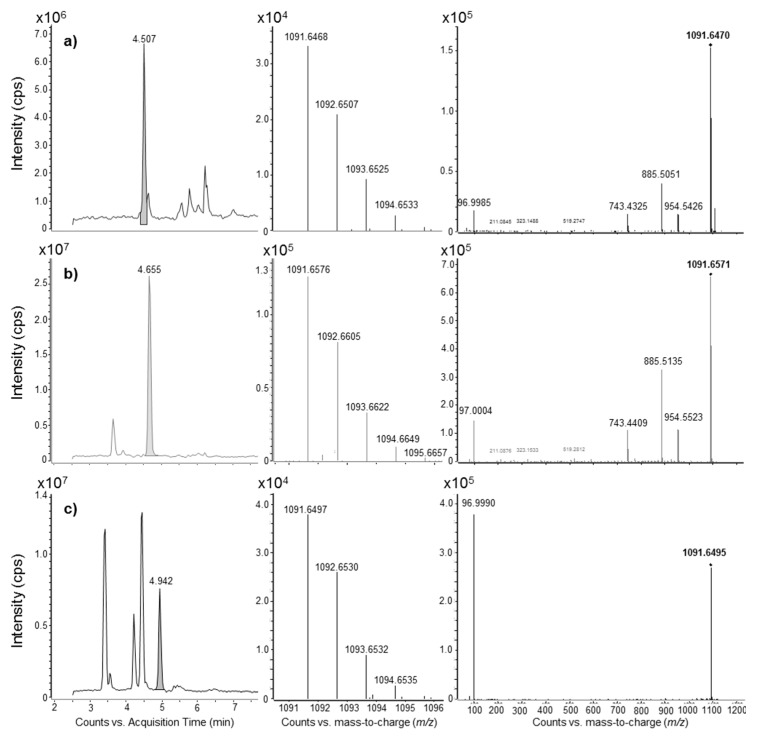
False positives for MTX2. Negative electrospray chromatogram, HRMS spectra and averaged HRMS/MS spectra (CEs = 25, 50 and 75 eV) of 1091.5 *m*/*z* found in crude extracts of: (**a**) *G. pacificus* G10-DC, (**b**) *Gambierdiscus* sp. Viet Nam and (**c**) *G. excentricus* Pulley Ridge Gam2.

MRM transitions of bi-charged anions (1637.5, 1648.2, 1656.0 *m*/*z*) of MTX2 towards the [HOSO_3_]^−^ (96.9 *m*/*z*) were not found in any of the strains examined. Putative MTX2 identified using 1091.5 and/or 1103.8 tri-charged anions as parent ions was found in *G. caribaeus*, *G. excentricus*, *G. pacificus* and *Gambierdiscus* sp. Viet Nam. HRMS analyses conducted on the most concentrated samples (*G. excentricus* strains, *G. pacificus* G10-DC and *Gambierdiscus* sp. Viet Nam) unveiled that peaks found in LRMS/MS for MTX2 were actually false positives: 1091.5 was indeed a mono-charged ion species, so it could not be the molecular tri-charged anion [M − 3H]^3−^ estimated for MTX2. HRMS analyses also revealed very similar *RT* and MS/MS spectra for the peak with a nominal mass of 1091.5 in the two strains *G. pacificus* G10-DC and *Gambierdiscus* sp. Viet Nam, even though they had a different accurate mass. Additionally, the compound with a similar nominal mass of 1091.5 had a different *RT* and HRMS/MS fragmentation pathway in the *G. excentricus* strain Pulley Ridge Gam2 (Figure 7).

**Figure 8:**
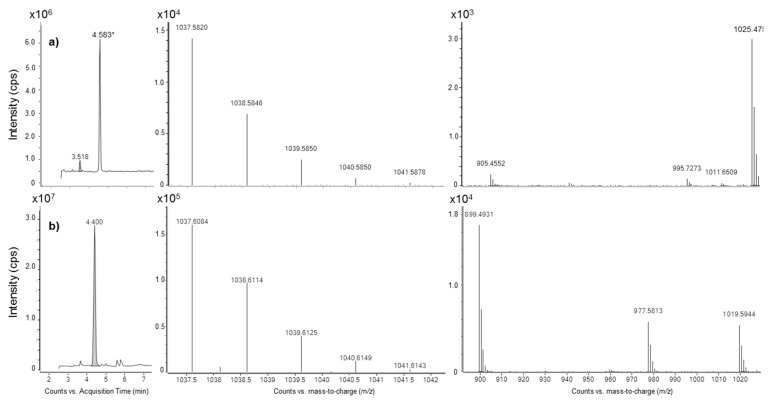
Putative candidates for MTX3. Negative electrospray chromatogram, HRMS spectra and averaged HRMS/MS spectra (CEs = 50, 100 and 150 eV) of 1037.5 *m*/*z* found in crude extracts of (**a**) *G. excentricus* VGO792, used as example for all *G. excentricus* strains; (**b**) *G. australes* S080911_1, used as example for all *G. australes*, *G. balechii*, *G. caribaeus*, *G. carpenteri*, *G. pacificus*, *G. scabrosus* strains. HRMS/MS spectra are presented with a zoom on an *m*/*z* range from 900 to 1020. * = peak only observed due to large mass window in quadrupole filter of HRMS/MS.

Putative MTX3 analogs were found in all *Fukuyoa* and *Gambierdiscus* strains examined. LRMS/MS already suggested at least two different compounds with *m*/*z* = 1037.6, i.e., one compound present in all *G. excentricus* strains (*RT* = 4.0–4.1 min) and one in some strains of other species (*RT* = 4.8–4.9 min) (Table 4). HRMS analyses confirmed the differences between *G. excentricus* strains and all the other strains (*G. australes* VGO1178, CCMP1653 and S080911_1; *G. balechii* VGO917 and VGO920; *G. caribaeus* CCMP1733 and Bill Hi Gam8; *G. carpenteri* GT4 and WHBR21; *G. pacificus* CCMP1650*; G. scabrosus* KW070922_1) (Figure 8).

For *G. excentricus* species, LH-20 fractionation of VGO791 and VGO792 showed no correlation between the cytotoxicity observed and the peak intensities corresponding to p-MTX2 (*V*_e_ = 21.0–34.0 mL) and p-MTX3 (*V*_e_ = 16.0–29.0 mL) analogs but only with MTX4 (Section 2.6).

## 3. Discussion

### 3.1. Gambierdiscus excentricus and the Discovery of Maitotoxin-4

Recent studies showed *G. excentricus* as one of the most toxic species known to date, both for CTXs and MTXs [61,69,75]. The species was first described in the Canary Islands [61], a subtropical region (North-Eastern Atlantic Ocean) from which Ciguatera Fish Poisoning (CFP) has recently been reported [76,77]. Subsequently, it has also been found in Brazil [78,79] and in the Caribbean Sea [75]. The aim of the present study was to identify maitotoxin or analogs produced by this species using high resolution mass spectrometry (HRMS).

Several difficulties had to be surmounted in the present study to identify such analogs. While the N2a cytotoxicity assay allowed for MTX detection at ng mL^−1^ levels, comparable to what had been reported by Caillaud, et al. [80], Q-Tof LC-HRMS (negative ion acquisition mode) had dramatically poorer sensitivity, with an LOD for MTX at 1.88 µg mL^−1^. The LOD of MTX using HRMS was relatively high compared to the LOD reported for LRMS [3]. However, as MTX itself was not present, an untargeted approach based on full-scan HRMS was necessary to screen for any potential analogs present, and the lower sensitivity of this technique had to be accepted. Compared to the need for µg quantities of toxin for LC-HRMS analysis, maitotoxic species only produce up to ca. 80 pg MTX eq cell^−1^ [69]. Hence, it was necessary to have a substantial biomass for purification purposes in order to obtain detectable amounts of toxin. *Gambierdiscus excentricus* is difficult to cultivate compared to other algae, including other *Gambierdiscus* species [69,75]. This is challenging because *Gambierdiscus* species grow extremely slowly (0.08–0.10 divisions day^−1^) compared to most other microalgae [69,75,81,82]. Hence, large-scale cultures of the Canary Island strains VGO791 and VGO792 were necessary to obtain sufficient material (>2 million cells) for LC-HRMS analysis.

Once sufficient biomass was obtained, toxicity screening (N2a-based assays) was applied to identify toxic fractions following liquid-liquid partitioning and LH-20 chromatography. Results of both N2a cytotoxicity and Ca^2+^ flux assays on LH-20 fractions suggested that *G. excentricus* strains produce compound(s) with relatively high molecular weight (early elution on LH-20) and Ca^2+^-related activity (N2a assays), similar to MTX. Chemical analyses using LC-HRMS (full scan mode) and LC-HRMS/MS (targeted mode) led to the discovery of a novel MTX analog, named maitotoxin-4 (MTX4), a sulfated compound with an accurate mono-isotopic mass of 3292.4860 Da (for the free acid form). No MTX2 was detected in any of the seven strains of *G. excentricus*, and, additionally, toxicity in LH-20 fractions was not correlated to putative MTX3. Therefore, and even though MTX4 was not yet completely purified, the correlation between MTX4 content in pre-purified fractions and their N2a cytotoxicity (Figure 6) supports the hypothesis that MTX4 is a major contributor to the toxicity of the MSF fraction of *G. excentricus*.

MTX equivalents measured in LH-20 fractions of both strains of *G. excentricus*, using the N2a cytotoxicity assay (Section 2.6), were 3.5-fold higher than the MTX4 content measured via LC-LRMS/MS for an equivalent number of extracted cells. This factor of 3.5 does not necessarily indicate that the toxin content is overestimated using the cytotoxicity assay as the following assumptions were made: (i) MTX4 exhibits the same toxicity as MTX and (ii) MTX and MTX4 have the same behavior in MS, i.e., that they have an equimolar response. The relative toxicity between the two molecules is not yet known, and neither are their ionization and fragmentation yields known in MS. Furthermore, as suggested by Lewis et al. [57] for several other *Gambierdiscus* species, additional analogs of MTX4 may be present in the same LH-20 fractions, albeit at lower concentration as we were not able to identify such analogs by HRMS.

### 3.2. Screening for the Presence of Other MTX Analogs

Another aim of the present study was to assess the diversity of previously reported MTX analogs produced by different strains and species of the genera *Gambierdiscus* and *Fukuyoa*. In order to achieve this goal, an LC-LRMS/MS method (API 4000 QTrap) was developed to screen for the presence of four MTX analogs in the extracts of two strains of *F. ruetzleri* and 42 strains representing 11 species, one ribotype and one strain of undetermined species (*Gambierdiscus* sp. Viet Nam) of *Gambierdiscus* spp. (Section 4.6.2).

Previous LC-LRMS/MS studies conducted by a group from the Cawthron Institute [59,64,66,71,72,73,74] evaluated the presence of MTX and putative MTX3 in a total of 32 strains of *Gambierdiscus* and *Fukuyoa*, i.e., two strains of *F.* cf. *yasumotoi*, 13 strains of *G. australes*, one strain of *G. belizeanus*, one strain of *G. carpenteri*, two strains of *G. cheloniae*, two strains of *G. honu*, six strains of *G. lapillus*, four strains of *G. pacificus* and one strain of *G. polynesiensis*. These authors reported that MTX was only present in 11 out of 13 *G. australes* strains (0.3–36.6 pg MTX cell^−1^), one originating from Cook Islands [71] and the other ten from Kermadec Islands [74]. In our present study, MTX was only detected in the one strain of *G. australes* (S080911_1) from Japan, at a concentration of 22.6 pg MTX cell^−1^, also confirmed by LC-HRMS. The other three strains of *G. australes* examined in this study (CCMP1653, from Hawaii; VGO1178 and VGO1181, from Canary Islands) did not contain detectable (>LOD) levels of MTX. This absence of MTX in the other strains may derive from comparatively high LODs around 1/10th of the concentration of MTX detected in the *G. australes* strain from Japan. Still, this finding is consistent with previous studies in suggesting that MTX itself has been mostly confirmed in strains of *G. australes*, at least since the most recent taxonomic separation into species and phylotypes. Again, chemical diversity in *G. australes* may also be larger than reported until now since Lewis et al. [57] reported at least two compounds with Ca^2+^ flux activity in a strain of *G. australes*.

Maitotoxin-4 was produced by all strains of *G. excentricus* examined in this study (13.0–72.8 pg MTX eq cell^−1^) (Table 4), independently of their geographical origin, albeit all seven from the Atlantic Ocean (Brazil, Canary Islands and Caribbean Sea), and was not detected in any other species examined. These findings suggest that the production of certain MTX analogs is likely to be species-specific within *Gambierdiscus* and *Fukuyoa* genera.

While results were clear and consistent with previous studies for MTX and MTX4, further examination is needed concerning MTX2 and MTX3. Maitotoxin-2 was described only once, in 1990, from an Australian strain of *Gambierdiscus* sp. (NQ1) by Holmes et al. [55]. To our knowledge, no LC-MS/MS method has been described for the detection of MTX2, set aside one study by Lewis et al. [56], which reported negative ionspray MS data based on infusion (using an orifice voltage of ‒120 V) of MTX2 dissolved in MeOH:H_2_O (1:1, *v*/*v*). The assigned negative ion species for the most intense MS peaks were: 1648.2 *m*/*z* for [M + Na − 3H]^2−^, 1656.0 *m*/*z* for [M + K − 3H]^2−^, 1098.6 *m*/*z* for [M + Na − 4H]^3−^ and 1103.8 *m*/*z* for [M + K − 4H]^3−^. MTX2 is assumed to be a mono-sulfated compound [53,56]. In the absence of MS fragmentation data for maitotoxin-2 (MTX2), the MRM transitions chosen in this study were based on bi-charged and tri-charged molecular anions and their respective sodium and potassium adducts pointing towards the hydrogenated sulfate anion [HOSO_3_]^−^. Therefore, analysis additionally included 1091.5 *m*/*z* for [M − 3H]^3−^ and 1637.5 *m*/*z* for [M − 2H]^2−^, derived from analogy with spectral behavior observed for MTX and MTX4. None of the strains presented a chromatographic peak with both bi- and tri-charged anion clusters in the mass spectrum. All strains of several species (*G. caribaeus*, *G. excentricus*, *G. pacificus* and *Gambierdiscus* sp. Viet Nam) were positive for the anion cluster of the nominal mass 1091.5 (which could potentially correspond to [M − 3H]^3−^ of MTX2). HRMS unveiled that these compounds were false positives of MTX2: the parent ion 1091.5 is actually a mono-charged ion species in *G. excentricus* strains, *G. pacificus* G10-DC and *Gambierdiscus* sp. Viet Nam (not sufficient sample material for *G. caribaeus*). Therefore, low resolution MS may misidentify these compounds for MTX2 in several species. Moreover, different retention time, accurate mass and HRMS/MS fragmentation pattern also revealed that the sulfated compound with an *m*/*z* 1091.5 found in *G. excentricus* strains was different from those found in *G. pacificus* G10-DC and *Gambierdiscus* sp. Viet Nam (Figure 7).

Maitotoxin-3 was isolated in 1994 from an Australian strain of Gambierdiscus sp. (WC1/1) by Holmes, et al. [53]. The only existing MS data are from positive ionspray MS analysis at different orifice potentials [56]. The authors described MTX3 as a disulfated compound (MW = 1060.5 Da for the disodium salt), with the most intense MS peak at 1039.5 *m*/*z* for the mono-sodiated adduct [M + Na]^+^ [56]. The only existing LC-LRMS/MS method for MTX3 detection has been developed by Kohli at the Cawthron Institute (Nelson, New Zealand) in 2013 and uses negative ionization mode [72]. This method identified a putative MTX3 (p-MTX3) based on the MRM transition *m*/*z* 1037.6 → 96.8. The parent ion at 1037.6 *m*/*z* had been incorrectly assigned to [M − H]^−^ by Kohli [72]; indeed, according to the original study [56], it actually corresponds to [M + Na − 2H]^−^. All but one (G. carpenteri Merimbula) out of the 32 strains examined by the group of the Cawthron Institute were positive for the presence of p-MTX3 [59,64,66,71,72,73,74]. All the strains examined in this present study were also positive for the presence of p-MTX3, suggesting that p-MTX3 is ubiquitous within Gambierdiscus and Fukuyoa genera. Interestingly, the parent ion chosen for p-MTX3 ([M + Na − 2H]^−^) has the same nominal *m*/*z* as that for 2,3-dihydroxyCTX3C in negative ionization MS after loss of one molecule of water (*m*/*z* 1037.5 for [M – H − H_2_O]^−^). 2,3-dihydroxyCTX3C is considered to be an oxidation product of CTX3C and has extensively been found in Gambierdiscus [80,83]. In one study, conducted by Roeder et al. [83], however, it had been the only analog present in ten out of a total of eleven Gambierdiscus strains, and this study also used low resolution negative ionization MS. It is surprising that CTX3C had not been detected in that study along with 2,3-dihydroxyCTX3C, except for one strain (Gambierdiscus sp. Viet Nam). Therefore, we presume that this study by Roeder et al. [83] may have misidentified 2,3-dihydroxyCTX3C, as it is likely that the actual compound present was the ubiquitous p-MTX3. Therefore, we recommend that when attempting to detect 2,3-dihydroxyCTX3C in negative mode LRMS/MS to verify that the compound does not have a loss of sulfate since this points towards p-MTX3 rather than 2,3-dihydroxyCTX3C. In our study, the tentative identification of p-MTX3 involved different possible parent ions (in some cases 1037.6, on other occasions 1015.5, and sometimes both) with different retention times, therefore different compounds were present that could be tentatively related to MTX3. HRMS/MS analyses for the most concentrated samples confirmed presence of sulfate ester group(s) in these compounds which is coherent with these compounds responding to the transition of the sulfate loss in low resolution tandem MS. At least one of the compounds, i.e., the one identified in all G. excentricus strains, does not correlate with cytotoxicity which suggests that this potential MTX analog is not important in accounting for toxicity.

It should also be noted that *Gambierdiscus* species produce other polyether compounds besides MTX containing sulfate ester group(s). Consequently, the presence of sulfate ester group(s) alone does not constitute definitive evidence for the presence of MTX. For example, a recent study conducted by Rodríguez, et al. [84] reported on the isolation and structural characterization (HRMS and NMR) of gambierone (C_51_H_76_O_19_S, with an exact mass of 1024.47015 Da) from *G. belizeanus* CCMP401. This molecule is a ladder-shaped polyether toxin and presents a sulfate ester group, but, contrarily to MTX, it behaves as a sodium channel activator such as CTXs, albeit with significantly smaller activity. Similarly, Watanabe, et al. [85] reported on the structural elucidation (HRMS and NMR) of gambieroxide (C_60_H_90_O_22_S, with an exact mass of 1194.5644), another sulfate-containing polyether compound isolated from *G. toxicus* GTP-2 (French Polynesia). Its chemical structure is very similar to that of yessotoxin (YTX); its biological activity has not yet been described.

Care should be taken in compound identification when operating in low-resolution mass spectrometry, especially when the standard is not commercially available and when searching for non-specific MRM transitions. In the present study, purification of MTX4 has not been completed and it was not possible to elucidate the full structure due to compound scarcity. Still, the evidence presented here for MTX4 as a MTX analog is based on (i) ion cluster similarity of MTX4 with MTX, (ii) targeted HRMS (loss of sulfate), (iii) bioguided fractionation behavior (both partitioning and size-exclusion chromatography), and (iv) the high cytotoxicity and Ca^2+^ influx activity. These data taken together strongly suggest that the novel molecule is a MTX analog. As MTX4 is a compound correlated with high cytotoxicity and could serve as a biomarker for the highly toxic *G. excentricus* species in the Atlantic area, further studies will focus on HRMS specific fragmentation pathways of MTX and MTX4, in both positive and negative ionization modes, and eventually nuclear magnetic resonance once sufficient compound is available.

## 4. Materials and Methods

### 4.1. Reference Toxins and Chemicals

Maitotoxin (MTX) was purchased from Wako Chemicals USA, Inc. (Richmond, VA, USA) and was used as the reference standard for cellular bioassays and chemical analyses. MTX was dissolved and stored in MeOH:H_2_O (1:1, *v*/*v*). The stock solution was prepared at a concentration of 20 µg mL^−1^.

HPLC grade methanol and dichloromethane for extraction were purchased from Sigma Aldrich (Saint Quentin Fallavier, France). Milli-Q water was supplied by a Milli-Q integral 3 system (Millipore, Saint-Quentin-Yvelines, France). Water (Optima quality), acetonitrile (Optima quality), formic acid (Puriss quality) and ammonium formate (Purity for MS) were used to prepare mobile phases. These chemicals were purchased from Sigma Aldrich (Saint Quentin Fallavier, France).

Eagle’s Minimum Essential Medium (EMEM, ATCC^®^ 30-2003) for culture of mouse neuroblastoma neuro-2a (N2a) cells at the Phycotoxins Laboratory was purchased from the American Type Culture Collection (ATCC). Roswell Park Memorial Institute 1640 medium supplemented with glutamine (RPMI-1640-GlutaMAX™) for culture of N2a cells at the ANSES Laboratory was purchased from Thermofisher Scientific (Waltham, MA, USA). The following additives to the N2a medium were purchased from Sigma Aldrich (Saint Quentin Fallavier, France): sodium pyruvate, streptomycin, penicillin and fetal bovine serum (Phycotoxins Laboratory) or fetal calf serum (ANSES Laboratory). N2a assay reagents were also purchased from Sigma Aldrich (Saint Quentin Fallavier, France): trypsin-(ethylenediaminetetraacetic acid) (trypsin-EDTA) and 3-(4,5-dimethylthiazol-2-yl)-2,5-diphenyl tetrazolium bromide (MTT). Fluo-4-AM and Hoechst 33342 probes for the N2a Ca^2+^ flux high-content screening (HCS) assay were purchased from Thermofisher Scientific (Waltham, MA, USA).

### 4.2. Gambierdiscus and Fukuyoa Strains Examined in This Study

The 44 strains of *Gambierdiscus* and *Fukuyoa,* which were examined in this study, and their location of origin, culture conditions and number of cells extracted are listed in Table 5. Strains were cultivated either at the Phycotoxins Laboratory (PHYC, Ifremer, Nantes, France) [69], or at the Center for Coastal Fisheries Habit Research Laboratory (CCFHR, NOAA, Beaufort, NC, USA) [75], or at the University of Rio (UNIRIO, Federal University of Rio de Janeiro State, RJ, Brazil) [79].

### 4.3. Cell Pellet Extraction

Cell pellets of *Gambierdiscus* and *Fukuyoa* strains were extracted three times with MeOH (30 mL per 1 million cells) using a 3 mm diameter probe sonicator (Q-Sonica, Q700, Newtown, CT, USA) at 30% of the total power (500 W). The sonication was conducted in an ice bath (0 °C) for 15 min in pulse mode (10 s ON, 5 s OFF). At the end of each sonication step, the supernatant (crude extract) was collected by centrifugation (4 °C, 10 min, 4000 *g*). Crude extracts were filtered through a Nanosep MF 0.2 μm filter and stored at −20 °C until LC-MS analyses.

### 4.4. Fractionation of Gambierdiscus excentricus VGO791 and VGO792

After three weeks of semi-continuous culture [69], cells were first filtered on a 25 µm sieve, then harvested by centrifugation (20 min, 3000 *g*, 4 °C) in 50 mL Falcon^®^ tubes. A total of 2.20 million cells (3.307 g wet pellet, 4.5 L of culture, 15 flasks) and 2.16 million cells (3.076 g wet pellet, 4.5 L of culture, 15 flasks) were harvested for *G. excentricus* VGO791 and VGO792, respectively. Cell pellets were stored at −20 °C until further extraction for toxicity screening and chemical analyses. After the cells had been harvested in log phase growth, they were extracted as described above (Section 4.3). An aliquot of crude extract volume was filtered through a Nanosep MF 0.2 μm filter and stored at −20 °C until cellular bioassays and LC-MS analyses. The remnant part was evaporated under N_2_ at 40 °C and stored at −20 °C.

#### 4.4.1. Liquid-Liquid Partitioning

The residues of crude extracts (0.263 g for *G. excentricus* VGO791 and 0.251 g for *G. excentricus* VGO792) were suspended in dichloromethane (50 mL per 1 million cells) and partitioned twice with MeOH:H_2_O (3:2, *v*/*v*) (25 mL per 1 million cells) as previously described by Satake, et al. [91]. Maitotoxins are supposed to partition into the aqueous methanol soluble fraction (MSF). Once the MSF was isolated, it was blown dry under N_2_ gas at 40 °C and stored at −20 °C. The dried MSF residue was re-dissolved in 1.5 mL MeOH:H_2_O (1:1, *v*/*v*) and filtered through a Nanosep MF 0.2 μm filter.

#### 4.4.2. Size-Exclusion Chromatography (SEC): Sephadex™ LH-20

Prior to use, Sephadex™ LH-20 powder (10 g) was swollen in methanol (MeOH) over one night, then gently packed in a glass open column (intern diameter: 1 cm) in one continuous motion and finally rinsed with MeOH. Bed height was 52.7 cm, hence bed volume was calculated to be 41.4 mL. An aliquot of MSFs of *G. excentricus* VGO791 (0.745 mL, 1.082 million cells, 32.5 mg MSF residue) and *G. excentricus* VGO792 (0.700 mL, 0.998 million cells, 29.9 mg MSF residue) were separately deposited on the top of the LH-20 column; compounds were then eluted with MeOH under atmospheric pressure (flow rate: 0.4 mL min^−1^). Eluting fractions were collected as follows: 50 fractions of 2.5 mL each for *G. excentricus* VGO791; 1st fraction of 10 mL, 45 fractions of 1 mL and two last fractions of 30 mL for *G. excentricus* VGO792. All the fractions were screened for toxicity (N2a assays), and analyzed by LC-MS at either high or low resolutions, in either HRMS full scan or targeted HRMS/MS and LRMS/MS modes.

### 4.5. Neuroblastoma Neuro-2a (N2a) Assays

#### 4.5.1. N2a Cytotoxicity Assay

The N2a cytotoxicity assay was performed at the Phycotoxins Laboratory (Ifremer, Nantes, France) using the protocol for MTX detection described by Caillaud, et al. [92].

The N2a cell line was obtained from the American Type Culture Collection (ATCC, CCL 131). N2a cells were grown and maintained as described by Hardison, et al. [93]. The assay was carried out in 96-well flat-bottom Falcon^®^ tissue culture plates with vacuum gas plasma treatment for cell adhesion (Dominique DUTSCHER SAS, Brumath, France). Plates were seeded with 30,000 N2a cells per well and were incubated for 24 h until they were >90% confluent at the bottom of each well. The MTX standard, controls and *G. excentricus* samples were added next and incubated for 2.5 h. The 6-point MTX standard curve for this assay ranged from 0.29 to 29,127 ng mL^−1^ per well. Controls included buffer wells to provide maximum survival estimates and wells with the addition of 3% MeOH (final concentration in well) to identify any cell mortality caused by the presence of MeOH used to dissolve the samples. The following sample aliquots (3 µL additions) were tested: MSF extracts of *G. excentricus* VGO791 and VGO792 and their respective fractions obtained by SEC (LH-20). Total well volume was hence 103 µL.

For each sample, six 10-fold serial dilutions were tested in three separate experiments and three replicate wells for each dilution were run for each experiment. Sigmoidal dose-response curves were plotted using the four-parameter logistic model (4PL) and EC_50_ values (cell eq mL^−1^) were calculated for each sample using SigmaPlot^®^ 12. Quantitation of MTX eq in the samples using the N2a cytotoxicity assay was calculated by converting EC_50_ values (cell eq mL^−1^) into toxin equivalent per cell (pg MTX eq cell^−1^) taking into account the EC_50_ value obtained from the MTX standard curve. Results were then converted in µg MTX eq multiplying for the initial number of cells extracted.

Cell viability was assessed after 2.5 h incubation using the quantitative colorimetric 3-(4,5-dimethylthiazol-2-yl)-2,5-diphenyl tetrazolium bromide (MTT) assay [94] using a Tecan Infinite^®^ M200 plate reader (Tecan Austria GmbH, Grödig, Austria) at 544 nm. The viability of cells treated with MTX standard or algal extracts was estimated relative to control wells in solvent vehicle (3% MeOH in N2a medium).

#### 4.5.2. N2a Calcium Flux Assay

The high-content screening (HCS) assay employed an automated epifluorescence microscopy and image analysis of cells in a microtiter plate format [95,96]. In the present study, HCS assay was used to measure Ca^2+^ influx induced by MTXs using the N2a cell line. The assay was performed at the Toxicology of Contaminants Unit, ANSES Laboratory (Fougères, France).

N2a cells (ATCC, CCL 131) were grown and maintained as described by Sérandour et al. [97]. The assay was carried out in Nunc 96-well thin bottom microplates (Thermo Scientific, Waltham, MA, USA). Each well was seeded with 33,000 N2a cells and plates were incubated for 24 h until they were >90% confluent at the bottom of each well. Next, two fluorescent dyes, Hoechst 33342 (3 µg mL^−1^) for cell nuclei staining and Fluo-4-AM (5 µM) for _i_Ca^2+^ staining, were added to each well and the plates were placed back in the incubator for 40 min.

Vehicle control solution for the assay consisted of N2a culture medium without fetal calf serum (FCS) containing 5% MeOH. The MTX standard curve for the assay ranged from 26.7 to 102,776 pg mL^−1^ in well (from 7.81 to 30,000 pM) and was prepared in FCS-free N2a culture medium containing 5% MeOH. The crude extract and partially purified extracts from *G. excentricus* VGO791 were prepared as follows: 0.05 mL of each sample were diluted in 0.95 mL of FCS-free N2a culture medium resulting in a solution with final MeOH content of 5%.

Six wells per plate were dedicated to vehicle control exposure to identify any non-specific flux of Ca^2+^ due to the presence of 5% MeOH. For each sample, three separate experiments were performed, and each experiment was run in three replicate wells. N2a medium was discarded from the wells, one-by-one, and immediately replaced with 100 µL of vehicle control, MTX standard or *G. excentricus* VGO791 samples just before the fluorescence measurement. Fluorescence (388 nm for cell nuclei, 488 nm for _i_Ca^2+^) was followed in real-time with one image per 1.5 min frame rate using an ArrayScan VTI HCS Reader (Thermo Scientific, Waltham, MA, USA) with 10× objective. Intracellular calcium signal was assessed after 4.5 min exposure and expressed as a fold of intensity compared to control treatment (5% MeOH in FCS-free N2a culture medium).

### 4.6. LC-MS Analyses

#### 4.6.1. LC-HRMS and HRMS/MS (Q-Tof 6550 iFunnel)

LC-HRMS analyses were performed at the Phycotoxins Laboratory (Ifremer, Nantes, France) using a UHPLC system 1290 Infinity II (Agilent Technologies, Santa Clara, CA, USA) coupled to a high resolution time-of-flight mass spectrometer Q-Tof 6550 iFunnel (Agilent Technologies, Santa Clara, CA, USA) equipped with a Dual Jet Stream^®^ electrospray ionization (ESI) interface operating in negative mode. Toxins were separated using a reversed-phase C_18_ Kinetex column (100 Å, 2.6 μm, 50 × 2.1 mm, Phenomenex, Le Pecq, France) with water (A) and 95% acetonitrile/water (B). The column oven and the sample tray temperatures were set at 40 °C and 4 °C, respectively. The flow rate was set at 0.4 mL min^−1^, the injection volume was set at 3 µL. Separation was achieved using the following mobile phase gradient: from 10 to 95% B in 10 min, plateau at 95% B for 2 min, return to the initial condition (10% B) in 0.1 min and a re-equilibration period (10% B) for 3.9 min. The chromatographic run lasted 16 min per analysis. The MTX standard used for LC-HRMS experiments was at a concentration of 20 µg mL^−1^ MeOH:H_2_O (1:1, *v*/*v*).

The conditions of the ESI source were set as follows: source temperature, 200 °C; drying gas, N_2_; flow rate, 11 mL min^−1^; sheath gas temperature, 350 °C; sheath gas flow rate, 11 mL min^−1^; nebulizer, 45 psig; capillary voltage, −3.5 kV; nozzle voltage, 500 V. The instrument was mass calibrated in negative ionization mode before each analysis, using the Agilent tuning mix. A mixture solution of reference mass compounds (purine, 2 mL L^−1^; HP-0921, 1 mL L^−1^; HP-1221, 1 mL L^−1^; HP-1821, 2 mL L^−1^; HP-2421, 2 mL L^−1^) in MeOH:H_2_O (95:5, *v*/*v*) was infused with an isocratic pump to a separate ESI sprayer in the dual spray source at a constant flow rate of 1.5 µL min^−1^. Purine and HP-0921 allowed for correction of the measured *m*/*z* throughout the batch.

Mass spectrum detection was carried out in full scan and targeted MS/MS mode in negative ion acquisition. The full scan acquisition operated at a mass resolution of 45,000 Full Width at Half Maximum (FWHM) over a mass-to-charge ratio (*m*/*z*) range from 100 to 3200 with a scan rate of 2 spectra s^−1^. The LOD (S/N ratio > 3) calculated for MTX (negative mode EIC of 1688.8013 *m*/*z*) was 1.88 µg mL^−1^. The targeted MS/MS mode was performed in a Collision Induced Dissociation cell using a mass resolving power of 45,000 FWHM over the scan range *m*/*z* from 50 to 3200 with a MS scan rate of 10 spectra s^−1^ and a MS/MS scan rate of 3 spectra s^−1^. Three different collision energies were applied to the precursor ions to obtain a good fragmentation pathway.

All the acquisition and analysis data were controlled by MassHunter software (Agilent Technologies, Santa Clara, CA, USA). Raw data were processed using the Molecular Feature Extraction (MFE) algorithm of the Agilent MassHunter Qualitative Analysis software, version B.07.00, service pack 1 (Agilent Technologies, Santa Clara, CA, USA). The algorithm performs all tasks related to “peak-picking” and thus allowed for identification of all sample components down to the lowest-level abundance (abundance cut-off set at 500 counts) and to extract all relevant spectral and chromatographic information. Data-mining was carried out to manage data complexity and to correlate MS data to toxicity.

#### 4.6.2. LC-LRMS/MS (API 4000 QTrap)

LC-LRMS/MS experiments to monitor specific MTX congeners in the methanolic extracts obtained from the strains listed in Table 5 were performed at the Phycotoxins Laboratory (Ifremer, Nantes, France) using a LC system (UFLC XR Nexera, Shimadzu, Japan) coupled to a hybrid triple quadrupole/ion-trap mass spectrometer API 4000 QTrap (SCIEX, Redwood City, CA, USA) equipped with a turboV^®^ ESI source. Toxins were separated using the same chromatographic conditions as described above (Section 4.6.1). The injection volume was set at 5 µL. Mass spectrum detection was carried out in negative ion acquisition mode using multiple reactions monitoring (MRM).

The MTX standard calibration range for the LC-LRMS/MS experiments consisted of nine concentrations ranging from 0.1 to 20 µg mL^−1^ MeOH:H_2_O (1:1, *v*/*v*). MRM experiments were established using the following source settings: curtain gas set at 25 psi, ion spray at −4.5 kV, a turbogas temperature of 300 °C, gas 1 and 2 set, respectively, at 40 and 60 psi, an entrance and declustering potential of −10 V and −210 V, respectively. The limit of detection (LOD) and the limit of quantification (LOQ) were determined with the ordinary least-squares regression data method [98,99] using the lowest 3 points from the calibration curves. The LOD was calculated as 3 times the standard deviation of the y-intercepts over the slope of the calibration curve; the LOQ was calculated as 10 times the standard deviation of the y-intercepts over the slope of the calibration curve [98,99].

The fragment ion monitored for all the MRM transition of the MTX-group of toxins was the hydrogenated sulfate anion [HOSO_3_]^−^ (*m*/*z* 96.9). The precursor ions were chosen according to data available in literature or provided in this study. Seven and six MRM transitions (*m*/*z*), were monitored for MTX and MTX4, respectively, to permit the best toxin identification (Table 6) with a dwell time of 80 msec. Quantification of MTX and MTX4 was operated using the MRM transition [M − 2H]^2−^ → [M − 2H]^2−^. MTX4 was quantified over the MTX calibration curve, assuming equal molar response and applying the same LOD and LOQ calculated for MTX. In the absence of MS/MS data on MTX2, precursor ions (*m*/*z*) were selected according to Lewis et al. [56]. Bi-charged and tri-charged molecular anions were also calculated and added to the MRM method (Table 6). For MTX3, the lack of MS data in negative ion acquisition mode did not allow the selection of precursor ions (*m*/*z*) which were already described in literature. In the present study, a MRM transition involving [M + Na − 2H]^−^ as precursor ion was selected according to a previous study conducted by Kohli et al. [72]. MRM transitions in this study also involved the corresponding molecular mono-charged anion [M − H]^−^ (calculated *m*/*z* 1015.5), its di-sodium adduct (calculated *m*/*z* 1057.5) and the molecular bi-charged anion [M − 2H]^2−^ (calculated *m*/*z* 507.3) as precursor ions (Table 6).

## Figures and Tables

**Table 1:** List of the three MTXs known to date with relevant chemical information. *Gambierdiscus* sp. GII-1 was isolated from Gambier Islands (French Polynesia); *Gambierdiscus* sp. NQ1 from Queensland (Australia) and *Gambierdiscus* sp. WC1/1 from Australia. FAB^−^: fast atom bombardment ionization in negative ion acquisition mode. IS^+^: ionspray ionization in positive ion acquisition mode. IS^−^: ionspray ionization in negative ion acquisition mode. UNKN: unknown.

Name	Abbr.	Formula	Mass (Da)	Structural Studies	Toxicity (i.p. LD_50_ in Mice, µg kg^−1^)	Source	Reference
Maitotoxin	MTX	C_164_H_256_O_68_S_2_Na_2_	3423.5811	IR, UV (λ_max_ = 230 nm) LRMS/MS: FAB^−^ NMR with complete stereochemistry	0.050	GII-1	[1,31,32,33]
Maitotoxin-2	MTX2	UNKN (mono-sodiated salt of a molecule containing one sulfate ester)	3298	UV (λ_max_ = 230 nm) LRMS: IS^+^, IS^−^, FAB^−^	0.080	NQ1	[55]
Maitotoxin-3	MTX3	UNKN (di-sodiated salt of a molecule containing two sulfate esters)	1060.5	UV (λ_max_ = 200, 235 nm) LRMS: IS^+^	UNKN	WC1/1	[53]

**Table 2:** List of the assigned negative HRMS ion species for MTX and MTX4. MTX4 spectra originate from the pre-purified LH-20 fraction (*V*_e_ = 15.0–16.0 mL) of *G. excentricus* VGO792. The *m*/*z* values in the table correspond to the accurate mono-isotopic *m*/*z*. ND: not detected. UNKN: unknown.

		MTX	MTX4
**Elemental formula** (free acid form)		C_164_H_258_O_68_S_2_	UNKN
**Retention time** (*RT*, min)		4.09 min	4.58 min
**Ion species** (accurate mono-isotopic *m*/*z*)	[M − 2H]^2−^	1688.8027 (Δppm: −0.8)	1645.2357
	[M + Na − 3H]^2−^	1699.7914 (Δppm: +0.5)	1656.2256
	[M + 2Na − 4H]^2−^	1710.7814 (Δppm: +1.1)	1667.2075
	[M − 3H]^3−^	1125.5334 (Δppm: −1.4)	1096.4889
	[M − 4H]^4−^	843.8989 (Δppm: −2.1)	ND

**Table 3:** Amounts of MTX4 present in crude extract from approximately 2.2 million cells of *G. excentricus* VGO791 and VGO792 and the amounts of toxin remaining after the subsequent liquid-liquid partitioning and LH-20 chromatography purification steps. The MTX4 estimates were obtained using multiple reactions monitoring (MRM) mode on LC-LRMS/MS. Quantification of MTX4 was based on the MRM transition 1646.2 → 1646.2 *m*/*z* ([M − 2H]^2−^ → [M − 2H]^2−^) using MTX as the reference standard, quantified using the MRM transition 1689.8 → 1689.8 *m*/*z* ([M − 2H]^2−^ → [M − 2H]^2−^). Amounts of MTX4 were therefore expressed in µg MTX eq, assuming an equimolar response of MTX4 and MTX in MS. * MSF: aqueous methanol soluble fraction.

Strain Name	Sample Name	MTX4 (µg MTX eq)	% Recovery (Liquid-Liquid Partitioning)	% Recovery (LH-20)
*Gambierdiscus excentricus* VGO791	Crude extract	160.1	100%	
	Liquid-liquid partitioning of crude extract			
	MSF *	137.4	85.8%	
	Size-exclusion chromatography (LH-20) of MSF sample			
	*V*_e_ = 12.5–15.0 mL	19.1		13.9%
	*V*_e_ = 15.0–17.5 mL	69.2		50.4%
	*V*_e_ = 17.5–20.0 mL	19.0		13.8%
	*V*_e_ = 20.0–22.5 mL	4.5		3.3%
	*V*_e_ = 22.5–25.0 mL	1.5		1.1%
	*V*_e_ = 25.0–27.5 mL	1.2		0.9%
*Gambierdiscus excentricus* VGO792	Crude extract	43.1	100%	
	Liquid-liquid partitioning of crude extract			
	MSF *	34.2	79.4%	
	Size-exclusion chromatography (LH-20) of MSF sample			
	*V*_e_ = 13.0–14.0 mL	0.6		1.7%
	*V*_e_ = 14.0–15.0 mL	4.6		13.5%
	*V*_e_ = 15.0–16.0 mL	10.3		30.1%
	*V*_e_ = 16.0–17.0 mL	6.2		18.0%
	*V*_e_ = 17.0–18.0 mL	3.3		9.6%
	*V*_e_ = 18.0–19.0 mL	1.8		5.3%
	*V*_e_ = 19.0–20.0 mL	1.2		3.4%
	*V*_e_ = 20.0–21.0 mL	0.8		2.5%
	*V*_e_ = 21.0–22.0 mL	0.6		1.9%
	*V*_e_ = 22.0–23.0 mL	0.6		1.7%
	*V*_e_ = 23.0–24.0 mL	<LOQ		
	*V*_e_ = 24.0–25.0 mL	<LOQ		
	*V*_e_ = 25.0–26.0 mL	<LOQ		

**Table 4:** Screening for the presence of MTX analogs in crude extracts of a total of 44 strains of *Gambierdiscus* and *Fukuyoa* using LC-LRMS/MS analysis. MTX4 content is expressed in pg MTX eq cell^−1^, assuming an equimolar response of MTX4 and MTX in MS. *RT*: retention time. ND: not detected. *: only one replicate available. LOD for MTX and MTX4 per sample is indicated in parenthesis and it is expressed in pg MTX eq cell^−1^. LODs were calculated according to the number of cells extracted for each strain (Table 5).

Species	Strain	MTX	p-MTX2		p-MTX3		MTX4
		pg MTX cell^−1^ ± SD (*n* = 3) *RT* = 4.64 min	1091.5 → 96.9	1103.8 → 96.9	1015.5 → 96.9	1037.6 → 96.9	pg MTX eq cell^−1^ ± SD (*n* = 3) *RT* = 5.05 min
*F. ruetzleri*	Gam1	ND (<2.97)	ND	ND	ND	*RT* = 4.89 & 5.74	ND (<2.97)
	WH55-Gam4	ND (<4.97)	ND	ND	ND	*RT* = 4.88	ND (<4.97)
*G. australes*	CCMP1653 (NOAA 24) (T39)	ND (<1.54)	ND	ND	ND	*RT* = 4.86	ND (<1.54)
	S080911_1	22.6 ± 0.5	ND	ND	ND	*RT* = 4.86	ND (<0.80)
	VGO1178	ND (<2.46)	ND	ND	ND	*RT* = 4.87	ND (<2.46)
	VGO1181	ND (<2.22)	ND	ND	ND	*RT* = 4.87	ND (<2.22)
*G. balechii*	VGO917	ND (<1.66)	ND	ND	*RT* = 4.10 & 4.21	*RT* = 4.80	ND (<1.66)
	VGO920	ND (<2.02)	ND	ND	*RT* = 3.94	*RT* = 4.86	ND (<2.02)
*G. belizeanus*	CCMP399 (NOAA2) (SB03)	ND (<3.51)	ND	ND	ND	*RT* = 4.80	ND (<3.51)
	Keys Gam1	ND (<7.79)	ND	ND	ND	*RT* = 4.80	ND (<7.79)
	ST1-F4	ND (<6.20)	ND	ND	ND	*RT* = 4.83	ND (<6.20)
*G. caribaeus*	CCMP1733 (NOAA11)	ND (<2.27)	*RT* = 3.64 & 4.28	*RT* = 3.64 & 4.28	ND	*RT* = 4.84	ND (<2.27)
	Bill Hi Gam8	ND (<5.79)	*RT* = 4.05 & 4.30	*RT* = 4.05 & 4.30	ND	*RT* = 4.83	ND (<5.79)
	CCMP1651 (NOAA20)	ND (<10.27)	*RT* = 4.27	*RT* = 4.27	ND	*RT* = 4.82	ND (<10.27)
	Dive 1 fa Gam1	ND (<1.69)	*RT* = 4.26	*RT* = 4.26	ND	*RT* = 4.83	ND (<1.69)
	Mexico Algae1 Gam1	ND (<5.19)	*RT* = 4.27	*RT* = 4.27	ND	*RT* = 4.80	ND (<5.19)
*G. carolinianus*	ETB Exp28 Gam10	ND (<10.49)	ND	ND	*RT* = 4.55, 6.11 & 6.21	*RT* = 4.89, 6.11 & 6.21	ND (<10.49)
	Greece Gam2	ND (<7.73)	ND	ND	*RT* = 4.54 & 6.21	*RT* = 4.86, 6.21 & 7.17	ND (<7.73)
	RROV5	ND (<5.47)	ND	ND	*RT* = 5.12 & 6.25	*RT* = 5.12 & 6.25	ND (<5.47)
*G. carpenteri*	GT4	ND (<7.27)	ND	ND	ND	*RT* = 4.86	ND (<7.27)
	Jamaica Algae2 Gam1	ND (<6.51)	ND	ND	ND	*RT* = 4.81	ND (<6.51)
	Pat Hi Jar7 Gam11	ND (<10.72)	ND	ND	ND	*RT* = 4.86	ND (<10.72)
	WHBR21	ND (<6.62)	ND	ND	ND	*RT* = 4.84	ND (<6.62)
*G. excentricus*	Pulley Ridge Gam 2	ND (<0.39)	*RT* = 5.68	ND	ND	*RT* = 4.01	22.9 *
	UNR-07	ND (<0.63)	*RT* = 5.64	ND	ND	*RT* = 4.05	16.0 ± 2.3
	UNR-08	ND (<1.45)	*RT* = 5.68	ND	ND	*RT* = 4.05	19.8 ± 6.4
	VGO1035	ND (<2.64)	*RT* = 5.68	ND	ND	*RT* = 4.01	13.0 *
	VGO790	ND (<3.73)	*RT* = 5.67	ND	ND	*RT* = 4.01	23.2 *
	VGO791	ND (<0.29)	*RT* = 5.67	ND	ND	*RT* = 3.71 & 4.01	72.8 ± 8.5
	VGO792	ND (<0.30)	*RT* = 5.68	ND	ND	*RT* = 3.68 & 3.99	20.0 ± 2.9
*G. pacificus*	CCMP1650 (NOAA 9) (MR1)	ND (<1.02)	*RT* = 5.33, 6.06, 6.24 & 6.82	*RT* = 5.33, 6.06, 6.24 & 6.82	ND	*RT* = 4.89	ND (<1.02)
	G10DC	ND (<0.66)	*RT* = 5.06	ND	*RT* = 3.90 & 4.15	-	ND (<0.66)
*Gambierdiscus* sp. ribotype 2	CCMP1655 (MQ2)	ND (<5.87)	ND	ND	ND	*RT* = 4.83	ND (<5.87)
	Mixed PR	ND (<6.49)	ND	ND	ND	*RT* = 4.90	ND (<6.49)
	St Maartens Gam10	ND (<5.52)	ND	ND	ND	*RT* = 4.90	ND (<5.52)
	SW Algae Gam1	ND (<8.38)	ND	ND	ND	*RT* = 4.81	ND (<8.38)
*G. scabrosus*	KW070922_1	ND (<0.83)	ND	ND	ND	*RT* = 4.80	ND (<0.83)
*G. silvae*	UNR-30	ND (<1.76)	ND	ND	*RT* = 5.93 & 6.13	*RT* = 3.22, 4.76, 5.35, 5.93, 6.13, 6.98	ND (<1.76)
	VGO1167	ND (<1.27)	ND	ND	-	*RT* = 4.83	ND (<1.27)
	VGO1180	ND (<1.52)	ND	ND	*RT* = 5.92 & 6.14	*RT* = 4.68, 5.92 & 6.14	ND (<1.52)
*Gambierdiscus* sp.	Viet Nam	ND (<0.37)	*RT* = 5.14	ND	*RT* = 7.02	*RT* = 7.64	ND (<0.37)
*G. toxicus*	GTT-91	ND (<5.49)	ND	ND	*RT* = 2.94	*RT* = 4.89	ND (<5.49)
	HIT-0	ND (<6.00)	ND	ND	*RT* = 2.92	*RT* = 4.89	ND (<6.00)
	HIT-25	ND (<8.56)	ND	ND	*RT* = 2.90	*RT* = 4.88	ND (<8.56)

**Table 5:** List of the two *Fukuyoa* and 42 *Gambierdiscus* strains examined in this study along with their species designation, geographical origin, culture collection of origin, culture conditions, number of cells extracted for toxin analysis and the references where the strains have been previously cited. CCFHR: National Oceanographic and Atmospheric Administration (NOAA), National Ocean Service, National Centers for Coastal Ocean Science, Center for Coastal Fisheries Habit Research (CCFHR), Beaufort, NC, USA; CCVIEO: Culture Collection of Harmful Microalgae of IEO (CCVIEO), Centro de Vigo, Vigo, Spain; IRTA: Investigación y tecnología agroalimentarias, Department of Agriculture, Government of Catalonia, Sant Carles de la Ràpita, Spain; KU: Kochi University (KU), Kochi, Japan; NCMA: Provasoli—Guillard National Center for Marine Algae and Microbiota (NCMA), Bigelow Laboratory for Ocean Sciences, East Boothbay, Maine, USA; UNIRIO: Rio de Janeiro State, Federal University (UNIRIO), Rio de Janeiro, RJ, Brazil; VNIO: Viet Nam National Institute of Oceanography (VNIO, VAST), Vinh Nguyen, Nha Trang, Viet Nam.

Species/Phylotype	Strain	Geographical Origin	Culture Collection	Culture Conditions	Number of Cells Extracted	Reference
*F. ruetzleri*	Gam1	Southwater Cay, Belize	CCFHR	CCFHR	215,190	[57,68]
	WH55-Gam4	Flower Garden Banks National Marine Sanctuary (West Bank), Northwestern Gulf of Mexico, United States of America	CCFHR	CCFHR	128,800	[75]
*G. australes*	CCMP1653 (NOAA 24) (T39)	Tern Island, Hawaii, United States of America	NCMA	PHYC	416,220	[58,86]
	S080911_1	Kutsu, Susaki, Kochi, Japan	KU	PHYC	798,285	[87]
	VGO1178	Punta Hidalgo, Tenerife, Canary Islands, Spain	CCVIEO	PHYC	260,388	[62,69]
	VGO1181	Punta Hidalgo, Tenerife, Canary Islands, Spain	CCVIEO	PHYC	288,608	[69]
*G. balechii*	VGO917	Manado, Celebes Sea, Indonesia	CCVIEO	PHYC	384,650	[60,88]
	VGO920	Manado, Celebes Sea, Indonesia	CCVIEO	PHYC	317,600	[60]
*G. belizeanus*	CCMP399 (NOAA2) (SB03)	St. Barthélemy Island, Caribbean, Territorial collectivity of Saint-Barthélemy	NCMA	PHYC	182,495	[57,58]
	Keys Gam1	Florida Keys, Florida, United States of America	CCFHR	CCFHR	82,125	[68]
	ST1-F4	St. Thomas, US Virgin Islands, United States of America	CCFHR	CCFHR	103,300	[68]
*G. caribaeus*	CCMP1733 (NOAA11)	Carrie Bow Cay, Belize, Caribbean, United States of America	NCMA	PHYC	282,450	[58]
	Bill Hi Gam8	Waikiki Beach, Honolulu, Hawaii, United States of America	CCFHR	CCFHR	110,600	[69]
	CCMP1651 (NOAA20)	Grand Cayman Island, Caribbean, Territory of United Kingdom	NCMA	CCFHR	62,300	[58]
	Dive 1 fa Gam1	Ft. Pierce, Florida, United States of America	CCFHR	CCFHR	378,000	[68]
	Mexico Algae1 Gam1	Cancun, Mexico	CCFHR	CCFHR	123,395	[68]
*G. carolinianus*	ETB Exp28 Gam10	Dry Tortugas, United States of America	CCFHR	CCFHR	61,005	[68]
	Greece Gam2	Crete, Greece	CCFHR	CCFHR	82,775	[69]
	RROV5	Puerto Rico, United States of America	CCFHR	CCFHR	116,985	[68]
*G. carpenteri*	GT4	Carrie Bow Cay, Belize	CCFHR	CCFHR	88,000	[57,68]
	Jamaica Algae2 Gam1	Ocho Rios, Jamaica	CCFHR	CCFHR	98,250	[68]
	Pat Hi Jar7 Gam11	Waikiki Beach, Honolulu, Hawaii, United States of America	CCFHR	CCFHR	59,680	[57,69]
	WHBR21	Flower Garden Banks National Marine Sanctuary (West Bank), Northwestern Gulf of Mexico, United States of America	CCFHR	CCFHR	96,720	[68]
*G. excentricus*	Pulley Ridge Gam 2	Pulley Ridge, Florida, United States of America	CCFHR	CCFHR	1,630,000	[75]
	UNR-07	Armação dos Búzios, Rio de Janeiro, Brazil	UNIRIO	UNIRIO	1,013,833	[79]
	UNR-08	Armação dos Búzios, Rio de Janeiro, Brazil	UNIRIO	UNIRIO	441,490	[79]
	VGO1035	Playa Las Cabras, La Palma, Canary Islands, Spain)	CCVIEO	PHYC	242,050	[61]
	VGO790	Punta Hidalgo, Tenerife, Canary Islands, Spain	CCVIEO	PHYC	171,711	[61]
	VGO791	Punta Hidalgo, Tenerife, Canary Islands, Spain	CCVIEO	PHYC	2,200,160	[61]
	VGO792	Punta Hidalgo, Tenerife, Canary Islands, Spain	CCVIEO	PHYC	2,159,997	[61]
*G. pacificus*	CCMP1650 (NOAA 9) (MR1)	Moorea, Society Islands, French Polynesia	NCMA	PHYC	630,175	[58]
	G10DC	Malaysia	IRTA	PHYC	965,040	[80]
*Gambierdiscus* sp. ribotype 2	CCMP1655 (MQ2)	Martinique, Caribbean, insular region of France	NCMA	PHYC	109,080	[89]
	Mixed PR	Puerto Rico, United States of America	CCFHR	CCFHR	98,560	[75]
	St Maartens Gam10	St. Maarteens, Kingdom of the Netherlands	CCFHR	CCFHR	116,000	[75]
	SW Algae Gam1	Southwater Cay, Belize	CCFHR	CCFHR	76,410	[75]
*G. scabrosus*	KW070922_1	Kashiwa-jima Island, Otsuki, Kochi, Japan	KU	PHYC	771,711	[63,87]
*G. silvae*	UNR-30	Brazil	UNIRIO	UNIRIO	362,877	Unpublished strain
	VGO1167	Punta Hidalgo, Tenerife, Canary Islands, Spain	CCVIEO	PHYC	502,080	[62]
	VGO1180	Punta Hidalgo, Tenerife, Canary Islands, Spain	CCVIEO	PHYC	421,430	[62]
*Gambierdiscus* sp.	Viet Nam	Cau Island, Binh Thuan, South China Sea, Viet Nam	VNIO	PHYC	1,726,949	[67,83,90]
*G. toxicus*	GTT-91	Teahupoo, Tahiti, French Polynesia	CCFHR	CCFHR	116,625	[70]
	HIT-0	Hitiaa, Tahiti, French Polynesia	CCFHR	CCFHR	106,650	[70]
	HIT-25	Hitiaa, Tahiti, French Polynesia	CCFHR	CCFHR	74,800	[70]

**Table 6:** List of the MRM transitions (*m*/*z*) for the four MTXs known to date (LC-LRMS/MS, API 4000 QTrap). MRM transitions of MTX and MTX4 were chosen according to HRMS data provided in this study. Quantification of MTX and MTX4 was operated using the MRM transition [M − 2H]^2−^ → [M − 2H]^2−^ (in bold). In the absence of MS/MS data on maitotoxin-2 (MTX2) and maitotoxin-3 (MTX3), putative MRM transitions were chosen based on their MS spectral peaks reported in literature and assuming that they share the same fragmentation behavior as that of MTX and MTX4. * = M refers to the free acid form, i.e., without the sodium salt(s) on sulfate ester group(s), e.g., M = C_164_H_258_O_68_S_2_ for MTX.

Compound	MRM Transitions (*m*/*z*) *		CE (eV)	CXP (eV)
MTX	**[M − 2H]^2−^/[M − 2H]^2−^**	**1689.8/1689.8**	−40	−15
	[M − 2H]^2−^/[HOSO_3_]^−^	1689.8/96.9	−125	−21
	[M + Na − 3H]^2−^/[HOSO_3_]^−^	1700.8/96.9	−125	−21
	[M + 2Na − 3H]^2−^/[HOSO_3_]^−^	1711.8/96.9	−125	−21
	[M − 3H]^3−^/[M − 3H]^3−^	1126.2/1126.2	−40	−15
	[M − 3H]^3−^/[HOSO_3_]^−^	1126.2/96.9	−125	−21
	[M − 4H]^4−^/[HOSO_3_]^−^	844.4/96.9	−125	−21
MTX2	[M − 2H]^2−^/[HOSO_3_]^−^	1637.5/96.9	−125	−21
	[M + Na − 3H]^2−^/[HOSO_3_]^−^	1648.2/96.9	−125	−21
	[M + K − 3H]^2−^/[HOSO_3_]^−^	1656.0/96.9	−125	−21
	[M − 3H]^3−^/[HOSO_3_]^−^	1091.5/96.9	−125	−21
	[M + Na − 4H]^3−^/[HOSO_3_]^−^	1098.6/96.9	−125	−21
	[M + K − 4H]^3−^/[HOSO_3_]^−^	1103.8/96.9	−125	−21
MTX3	[M − H]^−^/[HOSO_3_]^−^	1015.5/96.9	−125	−21
	[M + Na − 2H]^−^/[HOSO_3_]^−^	1037.6/96.9	−125	−21
	[M + 2Na − 3H]^−^/[HOSO_3_]^−^	1057.5/96.9	−125	−21
	[M − 2H]^2−^/[HOSO_3_]^−^	507.3/96.9	−125	−21
MTX4	**[M − 2H]^2−^/[M − 2H]^2−^**	**1646.2/1646.2**	−40	−15
	[M − 2H]^2−^/[HOSO_3_]^−^	1646.2/96.9	−125	−21
	[M + Na − 3H]^2−^/[HOSO_3_]^−^	1657.2/96.9	−125	−21
	[M + 2Na − 3H]^2−^/[HOSO_3_]^−^	1668.2/96.9	−125	−21
	[M − 3H]^3−^/[M − 3H]^3−^	1097.1/1097.1	−40	−15
	[M − 3H]^3−^/[HOSO_3_]^−^	1097.1/96.9	−125	−21

## Supplementary Materials

The following are available online at www.mdpi.com/1660-3397/15/7/220/s1, Figure S1. Sigmoidal dose-response curve of MTX standard on the neuroblastoma N2a cytotoxicity assay after 2.5 h exposure. Error bars represent assay variability (standard deviation, SD) measured by running extracts in three separate assays. In each assay, three separate wells were used for assaying each fraction. Figure S2. Sigmoidal dose-response curve of MTX standard on the N2a-based HCS assay for Ca^2+^ flux after 4.5 min exposure. Ca^2+^ flux was estimated measuring the fluorescence of the Fluo-4-AM dye at 488 nm. Fluo-4-AM fluorescence was expressed as a fold of intensity compared to vehicle control condition (5% MeOH in FCS-free N2a medium). Error bars represent assay variability (standard deviation, SD) measured by running extracts in three separate assays. In each assay, three separate wells were used for assaying each fraction.
