# Supplementary material for: Maitotoxin-4, a Novel MTX Analog Produced by Gambierdiscus excentricus
Source: Mar Drugs. 2017 Jul 11;15(7):220. doi: 10.3390/md15070220 (PMC5532662; doi:10.3390/md15070220)
Supplement: Supplementary file 1 [file marinedrugs-15-00220-s001.pdf]

Supplementary Materials:

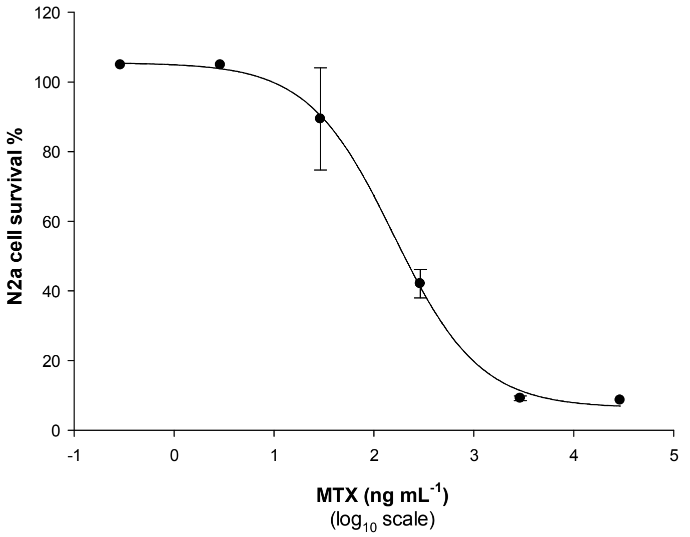

Figure S1. Sigmoidal dose-response curve of MTX standard on the neuroblastoma N2a cytotoxicity assay after 2.5 h exposure. Error bars represent assay variability (standard deviation, SD) measured by running extracts in three separate assays. In each assay, three separate wells were used for assaying each fraction.

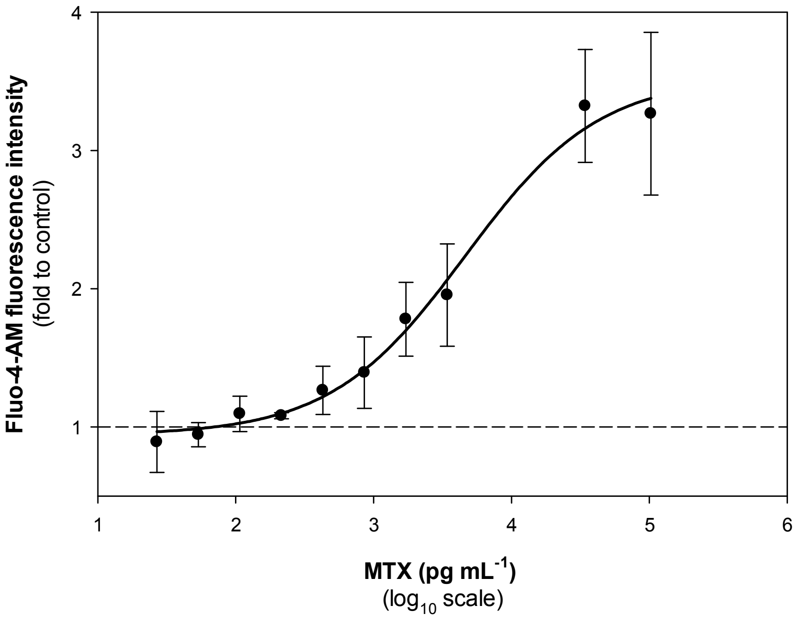

Figure S2. Sigmoidal dose-response curve of MTX standard on the N2a-based HCS assay for Ca^{2+} flux after 4.5 min exposure. Ca^{2+} flux was estimated measuring the fluorescence of the Fluo-4-AM dye at 488 nm. Fluo-4-AM fluorescence was expressed as a fold of intensity compared to vehicle control condition (5% MeOH in FCS-free N2a medium). Error bars represent assay variability (standard deviation, SD) measured by running extracts in three separate assays. In each assay, three separate wells were used for assaying each fraction.
